# Nonperturbative Fluorogenic Labeling of Immunophilins Enables the Wash-free Detection of Immunosuppressants

**DOI:** 10.1021/acscentsci.3c01590

**Published:** 2024-03-18

**Authors:** Marco Bertolini, Lorena Mendive-Tapia, Ouldouz Ghashghaei, Abigail Reese, Charles Lochenie, Anna M. Schoepf, Miquel Sintes, Karolina Tokarczyk, Zandile Nare, Andrew D. Scott, Stephen R. Knight, Advait R. Aithal, Amit Sachdeva, Rodolfo Lavilla, Marc Vendrell

**Affiliations:** † Centre for Inflammation Research, The University of Edinburgh, EH16 4UU Edinburgh, U.K.; ‡ IRR Chemistry Hub, Institute for Regeneration and Repair, The University of Edinburgh, EH16 4UU Edinburgh, U.K.; § Laboratory of Medicinal Chemistry, Faculty of Pharmacy and Food Sciences and Institute of Biomedicine UB (IBUB), University of Barcelona, Catalunya, Spain 08007; ∥ Concept Life Sciences Ltd, Edinburgh Bioquarter, Edinburgh EH16 4UX, U.K.; ⊥ Renal Transplant Unit, Queen Elizabeth Hospital, 1345 Govan Road, Glasgow G51 4TF, U.K.; # School of Chemistry, University of East Anglia, Norwich NR4 7TJ, U.K.

## Abstract

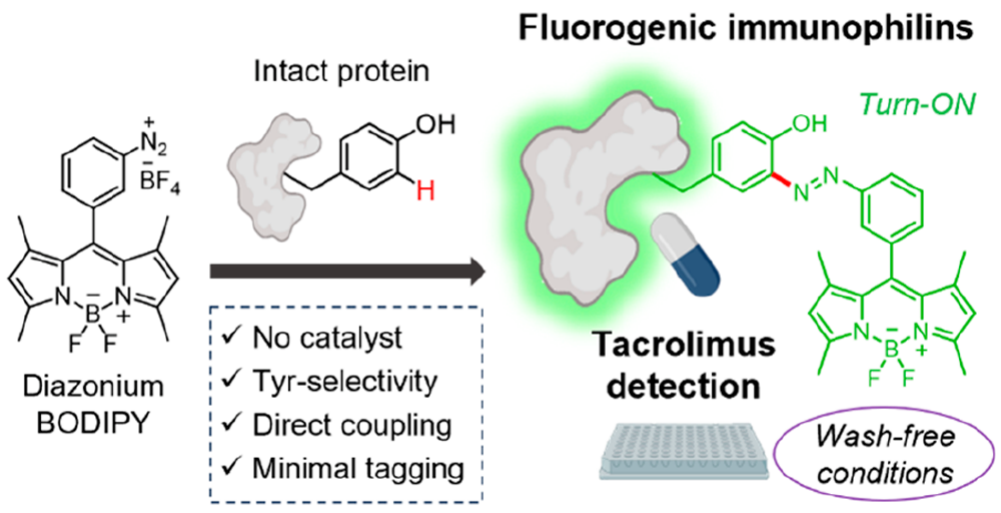

Immunosuppressants are clinically approved drugs to treat the potential rejection of transplanted organs and require frequent monitoring due to their narrow therapeutic window. Immunophilins are small proteins that bind immunosuppressants with high affinity, yet there are no examples of fluorogenic immunophilins and their potential application as optical biosensors for immunosuppressive drugs in clinical biosamples. In the present work, we designed novel diazonium BODIPY salts for the site-specific labeling of tyrosine residues in peptides via solid-phase synthesis as well as for late-stage functionalization of whole recombinant proteins. After the optimization of a straightforward one-step labeling procedure for immunophilins PPIA and FKBP12, we demonstrated the application of a fluorogenic analogue of FKBP12 for the selective detection of the immunosuppressant drug tacrolimus, including experiments in urine samples from patients with functioning renal transplants. This chemical methodology opens new avenues to rationally design wash-free immunophilin-based biosensors for rapid therapeutic drug monitoring.

## Abstract 2

We describe diazonium BODIPYs for the straightforward labeling of Tyr-containing peptides and whole proteins and the first fluorogenic analogue of immunophilin FKBP12 for the wash-free detection of tacrolimus in clinical biosamples.

## Introduction

Immunosuppressive drugs are widely used in clinics to avoid allograft rejection in patients undergoing organ transplantation.^1^ Importantly, real-time monitoring of the systemic levels of immunosuppressants (e.g., tacrolimus) is essential because their concentrations need to be adjusted within a narrow therapeutic window for appropriate dosage.^2^ For instance, reduced levels of immunosuppressants (i.e., in the low mM range) can lead to rejection of the transplanted organ, while excessive amounts result in medication-associated side effects, renal dysfunction, and an increased risk of infections.^3^ Immunoassays have been described as a strategy to optimize the dosage of immunosuppressive drugs.^4^ These assays are sensitive to low concentrations of the drug but are hampered by antibody cross-reactivity, batch-to-batch reproducibility, and the need for multistep labeling approaches.^5,6^ Label-free strategies (e.g., HPLC–MS analysis) can also be used for therapeutic drug monitoring, but those typically require sample preparation and have limited temporal resolution.^7^

With most optical technologies for therapeutic drug monitoring relying on antibody-based assays,^8,9^ there are few examples of fluorescent probes to detect immunosuppressive drugs in biosamples.^10−13^ In this regard, the site-specific labeling of peptides and proteins with fluorogenic dyes can be an effective strategy to generate turn-on probes for real-time analyte detection.^14−22^ Thus, we envisioned that the synthesis of fluorogenic reporters based on whole immunophilins may represent a modular and versatile approach to detect immunosuppressive drugs in real time. Our group and others have reported fluorogenic probes to measure immune cell activity^23−26^ by flanking enzyme-responsive substrates with fluorophore-quencher pairs^27−29^ and by embedding environmentally sensitive BODIPY amino acids within targeted peptide sequences.^30^ The preparation of these fluorogenic probes often requires solid-phase peptide synthesis (SPPS),^31−34^ which is not compatible with large proteins; in this work, we present a chemical strategy to prepare the first examples of fluorogenic immunophilins for the therapeutic monitoring of immunosuppressants.

The incorporation of fluorophores into proteins typically involves the derivatization of lysines (Lys) or cysteines;^35−37^ however, our group and others have shown that the labeling of aromatic residues (e.g., phenylalanine (Phe),^38^ tryptophan (Trp)^39−42^) can retain the bioactivity profile of native sequences.^43^ In the present work, we designed a chemical approach to selectively label tyrosine (Tyr) residues of immunophilins (e.g., FKBP12) with BODIPY fluorophores and applied them as fluorogenic probes for the detection of tacrolimus. This strategy is in principle applicable to different immunophilins and holds the potential to accelerate the rational design of fluorescent immunosensors for bioanalytical applications.

## Results and Discussion

### Chemical Synthesis and Optimization of Diazonium BODIPY Fluorogens for Tyr Labeling

Immunophilins are a family of peptidylprolyl isomerase proteins with a high affinity for immunosuppressive drugs. Among all immunophilins reported to date, FK506-binding protein (FKBP12) and peptidylprolyl isomerase A (PPIA) are known to bind the clinically approved drugs tacrolimus and cyclosporin A, respectively.^44,45^ Given their high binding affinity, we decided to employ this family of proteins for the rational design of protein-based fluorogenic probes for the wash-free detection of clinically approved immunosuppressants.

**Figure 1:**
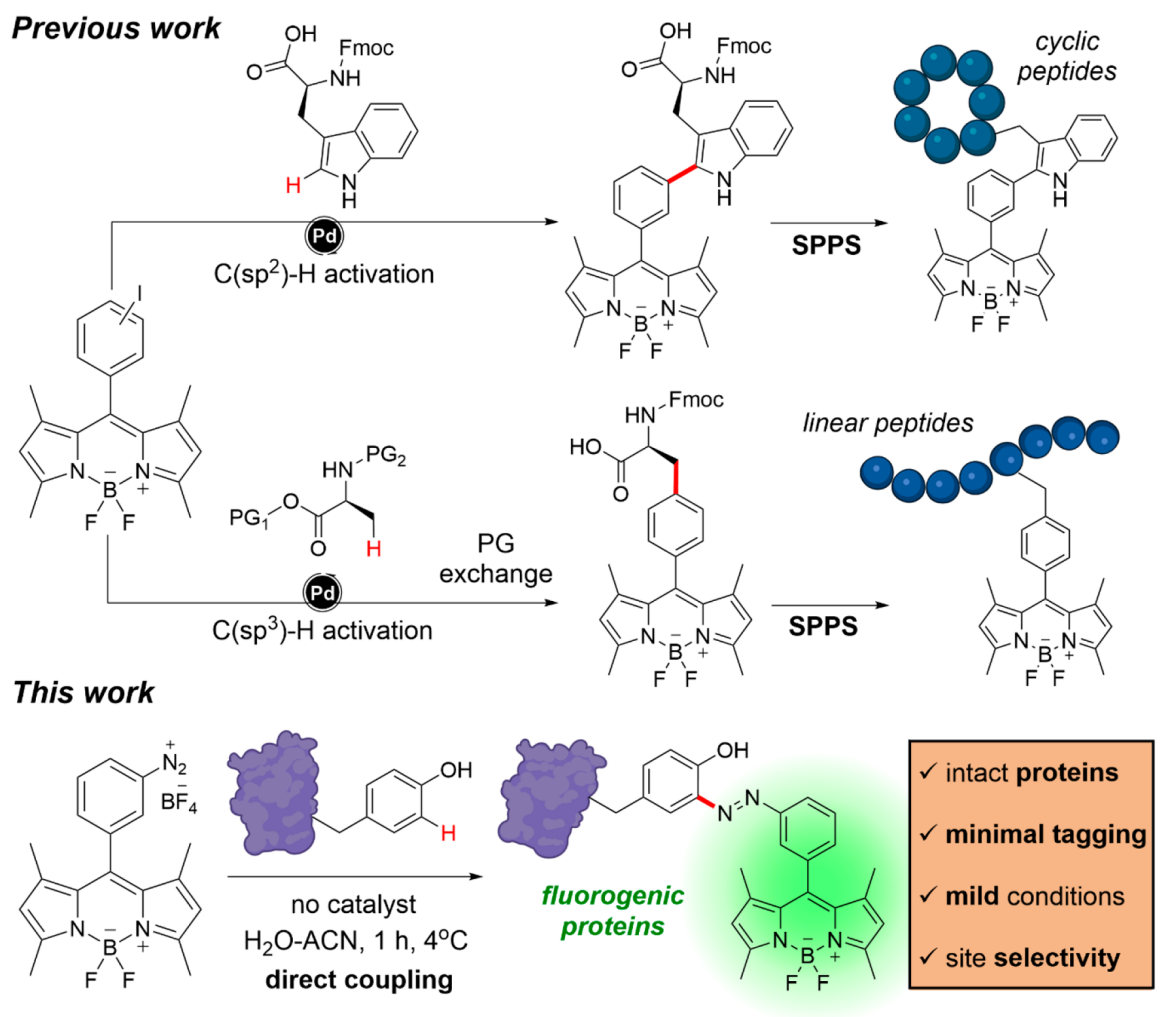
Fluorogenic labeling of peptides and proteins. Trp- and Phe-BODIPY amino acids can be synthesized by palladium-catalyzed C–H activation and subsequently incorporated into peptides by SPPS. Diazonium BODIPYs can selectively label tyrosine residues in whole immunophilins under mild conditions to produce fluorogenic biosensors. PG: protecting group.

Our group has optimized linker-free strategies to couple BODIPY fluorophores to aromatic amino acids (i.e., Trp or Phe)^38,46^ and subsequently employed them for the generation of fluorogenic peptides with turn-on emission properties (Figure 1);^40−42^ however, these building blocks cannot be readily incorporated into full protein structures, primarily due to the lack of aminoacyl-tRNA synthetases that recognize bulky fluorophores. Alternatively, we aimed to synthesize reactive BODIPY fluorogens that could site-specifically label proteins under physiological conditions. The group of Barbas III and others reported diazonium salts as suitable reagents to modify Tyr-containing proteins with chemical selectivity over other residues.^47−49^ This approach has been reported for the Tyr-directed modification of several biomolecules, including peptides and proteins,^50−57^ but it has not yet been reported as an effective means for direct BODIPY functionalization. Building on this reactivity profile, we designed the synthesis of stable diazonium BODIPY salts and subsequently employed them for the derivatization of Tyr amino acids in short peptides and whole proteins. We envisioned that this technology would allow us to (1) selectively attach small fluorogenic tags on Tyr residues in a one-step straightforward process and (2) avoid the disruption of the biomolecular recognition features of native peptides and proteins by means of a minimal azo bond linkage.

**Figure 2:**
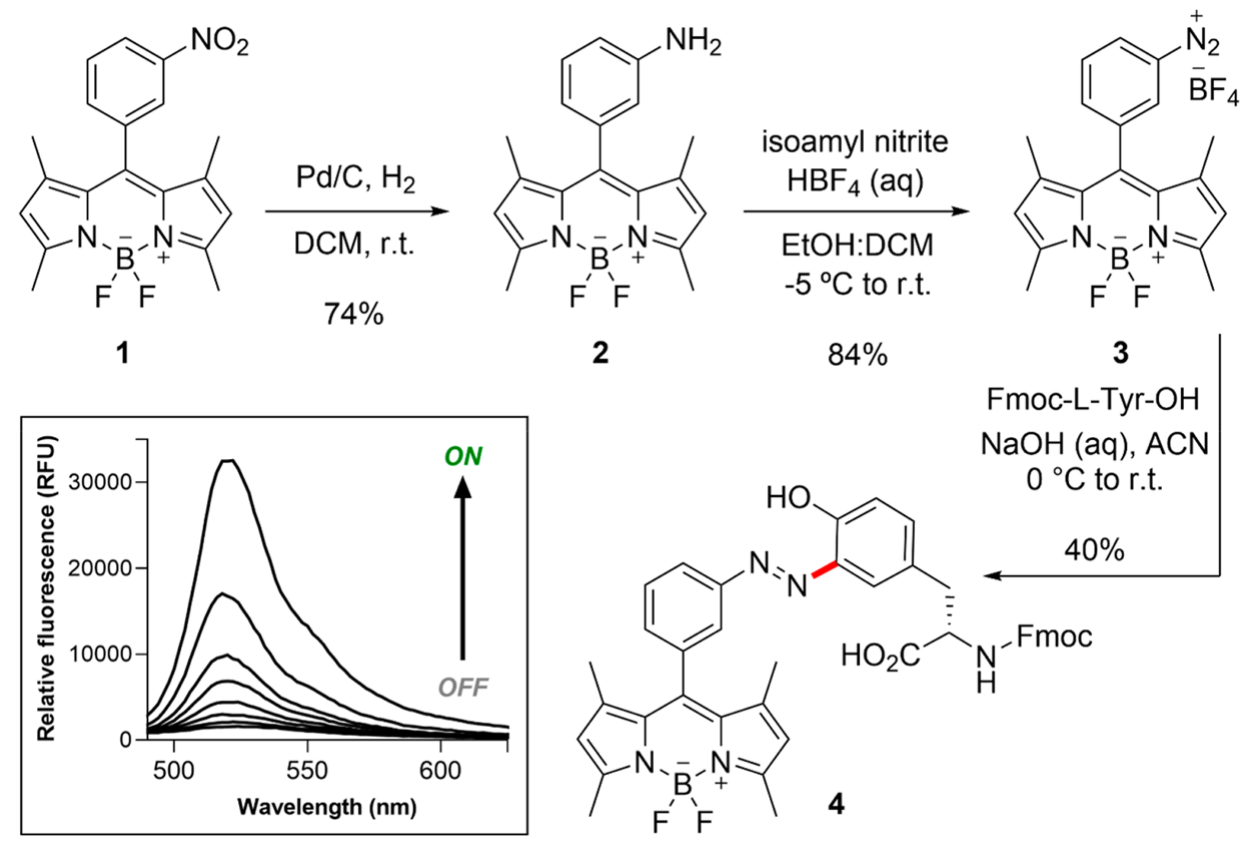
Synthesis of diazonium BODIPY and Tyr-BODIPY adducts. Synthetic scheme for the preparation of Tyr-BODIPY amino acids. (Left) Representative fluorescence spectra of compound **4** (25 μM) in phosphate buffer saline (PBS) and after incubation with increasing concentrations of phospatidylcholine liposome suspensions (from 3.75 to 0.004 mg mL^–1^) in PBS (excitation: 450 nm).

We designed the chemical synthesis of diazonium BODIPY analogues from the corresponding anilines, which were, in turn, obtained by the reduction of the nitro-functionalized derivatives (Figure 2). Nitro-BODIPY (**1**, Figure 2) and aniline BODIPY (**2**, Figure 2) were synthesized by the adaptation of reported procedures.^58^ Next, we made several attempts to obtain the diazonium BODIPY compound (**3**, Figure 2) by screening a variety of nitrite sources and acidic media. Initial attempts with sodium nitrite and hydrocloric acid or BF_3_·Et_2_O were not successful; instead, we found that the diazotization of compound **2** in aqueous HBF_4_ with isoamyl nitrite afforded the desired diazonium salts by simple precipitation and in yields >70% (Figure 2 and Figure S1). Of note, this synthetic strategy is scalable to hundreds of milligrams, and we confirmed that the diazonium salts can be safely stored as solids in sealed containers and under an inert atmosphere for over 2 years at −20 °C. To the best of our knowledge, this is the first example of an unsubstituted diazonium BODIPY fluorophore as a building block for the direct derivatization of peptides and proteins.

Next, we assessed the reaction of the diazonium BODIPY derivative with Fmoc-l-Tyr-OH in an aqueous medium (Figure 2). We observed good conversion rates at relatively short reaction times (e.g., around 1 h) under basic conditions using mixtures of H_2_O and ACN, and Tyr-BODIPY adduct **4** was isolated in yields of around 40% (Figure 2). Furthermore, we corroborated that the basic conditions used in the coupling of diazonium BODIPYs did not cause any epimerization of the asymmetric carbon of l-Tyr-OH (Figure S2), which confirms the suitability of this approach for labeling peptides and proteins without affecting their chirality. In view of the good reactivity of diazonium BODIPYs against Tyr residues, we measured the optical properties of the Tyr-BODIPY adduct **4** and observed extinction coefficients and excitation/emission wavelengths similar to those of BODIPY dyes (e.g., 500 and 520 nm, respectively) in the green region of the visible spectrum (Figures S3 and S4). Interestingly, Tyr-BODIPY amino acid **4** displayed notable fluorogenicity, with a >20-fold fluorescence enhancement in increasing concentrations of phosphatidylcholine-based liposomes (Figure 2) and in dioxane (Figure S5), with very low background fluorescence emission in aqueous media.

### Site-Specific Fluorogenic Labeling of Tyr-Containing Peptides

**Figure 3:**
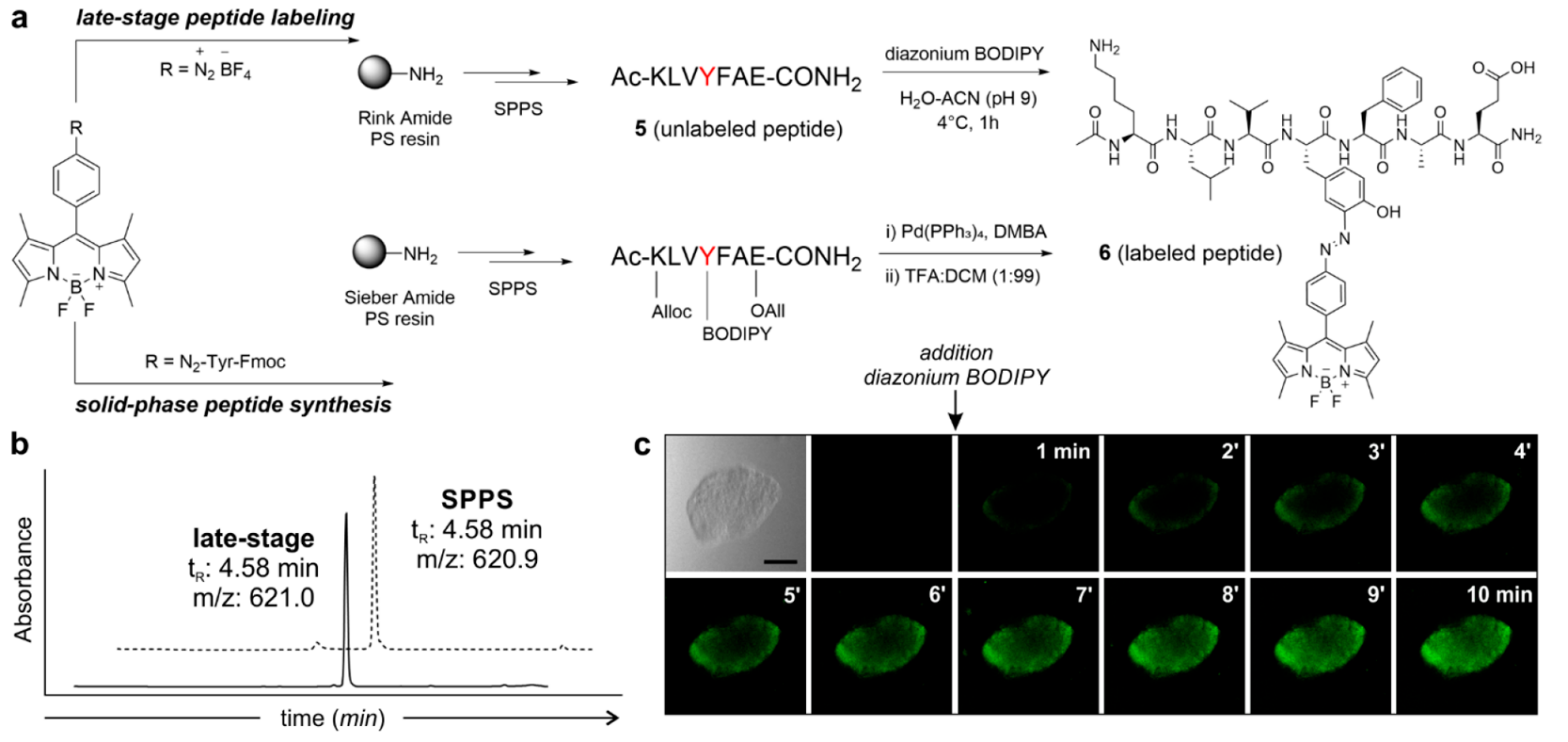
Fluorogenic labeling of peptides with diazonium BODIPY and Tyr-BODIPY. (a) Parallel synthetic strategies including late-stage derivatization of unprotected KLVYFAE (top) and SPPS using Fmoc-Tyr(BODIPY)–OH (bottom). (b) HPLC–MS traces of purified peptides using the two different synthetic approaches. (c) Representative time-course bright-field and fluorescence microscopy images (Movie S1 in Supporting Information) of self-aggregates formed by peptide **5** (100 μM) before and after incubation with diazonium BODIPY (5 μM) under wash-free imaging (excitation: 488 nm). Scale bar: 100 μm.

Given the properties of diazonium BODIPY as a reactive fluorogen for the derivatization of Tyr residues, we studied its application for labeling short peptides as simple model substrates. First, we analyzed the suitability of diazonium BODIPY for the late-stage modification of heptapeptide KLVYFAE (**5**, Figure 3). The KLVYFAE sequence is derived from the hydrophobic core of the β-amyloid protein (residues 16–22) and exhibits self-association into highly organized structures,^59^ thus being a suitable model peptide to corroborate the fluorogenic behavior of Tyr-BODIPY given the inherent ability of KLVFYAE to form self-aggregates in aqueous media.

First, we employed conventional SPPS procedures to prepare the peptide KLVYFAE (150 mg scale, >95% purity; see Supporting Information for full synthetic details and characterization data). Using dynamic light scattering, we confirmed that the sequence formed aggregates in a concentration- and time-dependent manner (Figure S6). Next, we employed the free peptide (i.e., without any side-chain protecting groups) for late-stage BODIPY derivatization. Reactions were conducted under basic conditions at 4 °C for 1 h, employing a slight excess (i.e., 1.2 equiv) of para-substituted diazonium BODIPY (compound **9**, see Supporting Information). Finally, the labeled peptide was purified by semipreparative HPLC to yield KLVY(BODIPY)FAE (**6**, Figure 3) with purities >95%.

In order to confirm the identity of the BODIPY-labeled peptide **6**, we synthesized the same sequence using Fmoc-Tyr(BODIPY)–OH as a building block for SPPS. Given the lability of the BODIPY fluorophore to acidic media, we used Fmoc-Lys(Alloc)–OH and Fmoc-Glu(OAll)–OH as amino acids with Pd^0^-labile protecting groups and an acid-labile Sieber Amide polystyrene resin. We performed the synthesis with conventional SPPS procedures (e.g., DIC and OxymaPure for couplings, piperidine:DMF (2:8) for Fmoc removal, and TFA:DCM (1:99) for solid support cleavage) and isolated the BODIPY-modified peptide by semipreparative HPLC with purities >95%. Importantly, we verified that the two batches of BODIPY-labeled peptide **6** (i.e., late-stage derivatization vs SPPS) were identical by HPLC–MS (Figure 3), thus demonstrating that the conjugation of diazonium BODIPY took place exclusively at the Tyr residue.

Having checked the suitability of diazonium BODIPY as a reagent for the derivatization of Tyr-containing peptides, we analyzed its potential as a reporter for real-time fluorescence imaging. For this purpose, we induced the formation of peptide **5**-based aggregates and incubated them at pH 9 with diazonium BODIPY to visualize them with fluorescence microscopy. As shown in Figure 3c, the diazonium BODIPY brightly labeled the aggregates in minutes under wash-free conditions. As a negative control, we attempted the same labeling experiment under neutral conditions (pH 7, Figure S7); however, lower fluorescence signals were detected, which confirmed that Tyr-specific conjugation of the diazonium BODIPY led to enhanced turn-on emission due to restricted rotation once embedded in the aggregated peptide. Altogether, these results confirmed the suitability of Tyr-BODIPY as a building block for the fluorogenic derivatization of peptide sequences.

### BODIPY-Labeled Immunophilins Enable Wash-free Detection of the Immunosuppressive Drug Tacrolimus

**Figure 4:**
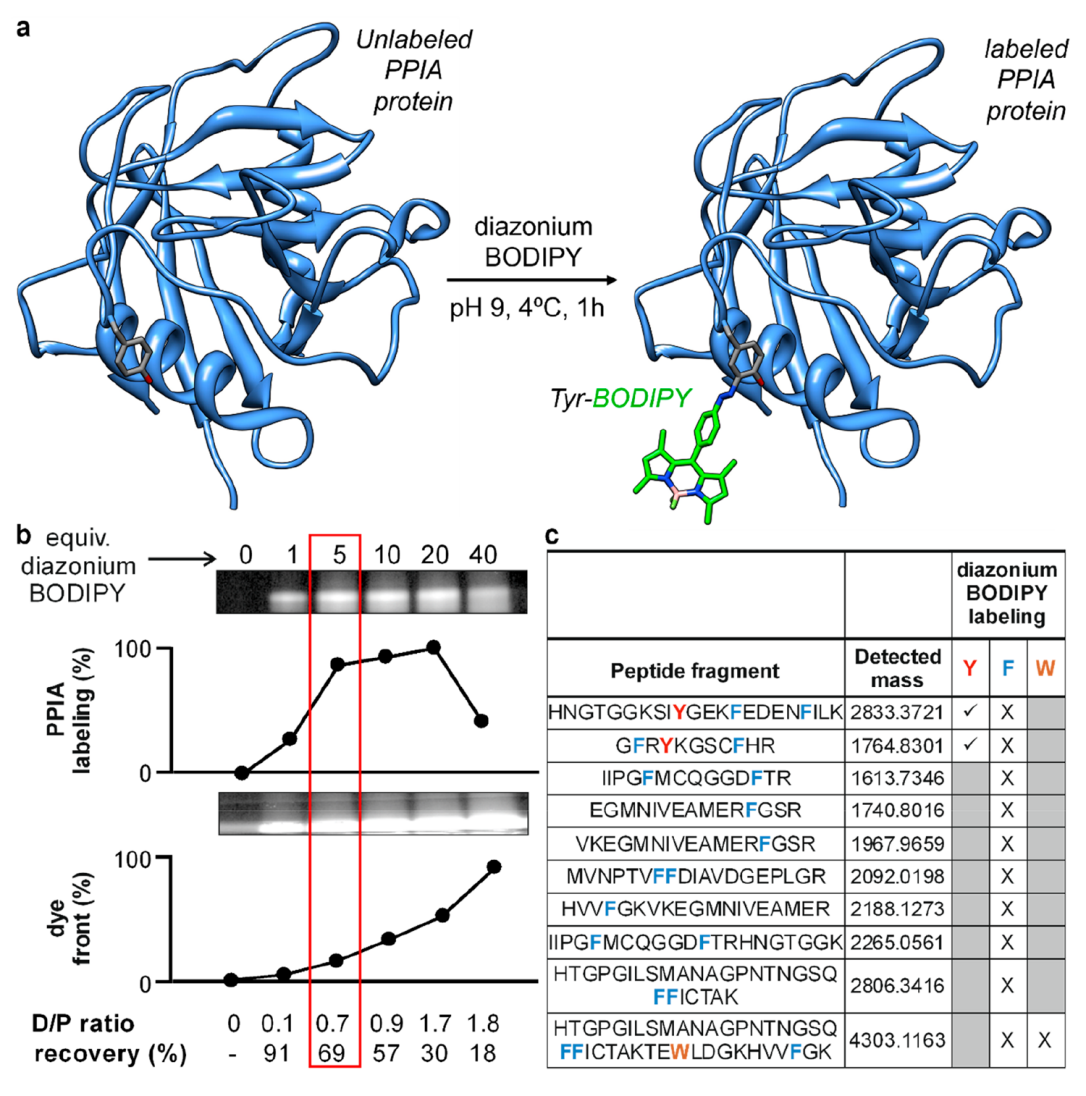
Optimization of whole immunophilin protein labeling using diazonium BODIPYs. (a) Tyrosine conjugation of the diazonium BODIPY to the immunophilin PPIA (PDB code: 1CWA). For simplicity, only one of the two native Tyr residues within the PPIA protein is illustrated. (b) In-gel fluorescence analysis of PPIA labeling reactions with different equivalents of diazonium BODIPY. D/P ratio: dye/protein ratio. (c) Peptide fragments identified by proteomic analysis after trypsin digestion of labeled PPIA. Residues detected by mass spectrometry are highlighted with the symbol √ whereas nondetected sequences are highlighted with the symbol ×.

Building on the results of BODIPY labeling in short peptides, next we optimized the direct coupling of diazonium BODIPY to immunophilins. For these experiments, we first employed commercially available immunophilin PPIA (peptidylprolyl isomerase A) as a model protein. PPIA is an 18 kDa protein with two Tyr residues in its primary sequence (e.g., Tyr47 and Tyr78); therefore, we envisaged that PPIA would be a suitable model immunophilin to assess the site selectivity of diazonium BODIPYs via fluorescence and proteomic experiments. First, we subjected PPIA to a series of conjugation reactions with increasing amounts of diazonium BODIPY (i.e., from 1 to 40 equiv) for 1 h at 4 °C. Successful BODIPY labeling of PPIA was verified by SDS-PAGE analysis and fluorescence scanning (Figure 4 and Figure S8). Although the labeling reactions proceeded to some extent at equimolar concentrations, the formation of bright conjugates was optimal when using 5 equiv of the fluorophore (Figure 4). Interestingly, a large excess of diazonium BODIPY did not result in brighter protein bands and led to the formation of precipitates with reduced recovery yields. Following the removal of unreacted dye by ultrafiltration, we measured the degree of labeling (DoL) for all of the conditions and obtained DoL values of around 1 when 5 equiv of diazonium BODIPY was used (Figure 4). We also examined the site selectivity of the diazonium BODIPY conjugation by the analysis of the labeled PPIA using mass spectrometry. Importantly, we confirmed BODIPY labeling at Tyr residues (Figure 4), and no conjugation to other aromatic amino acids (e.g., Phe, Trp) was detected (Figures S9 and S10). These results align with the preferential coupling of diazonium salts on tyrosines over more hindered tryptophans and histidines in a protein context.^47,57^ Altogether, these results validated the utility of diazonium BODIPY for the direct labeling of Tyr residues in whole immunophilins using a straightforward procedure.

**Figure 5:**
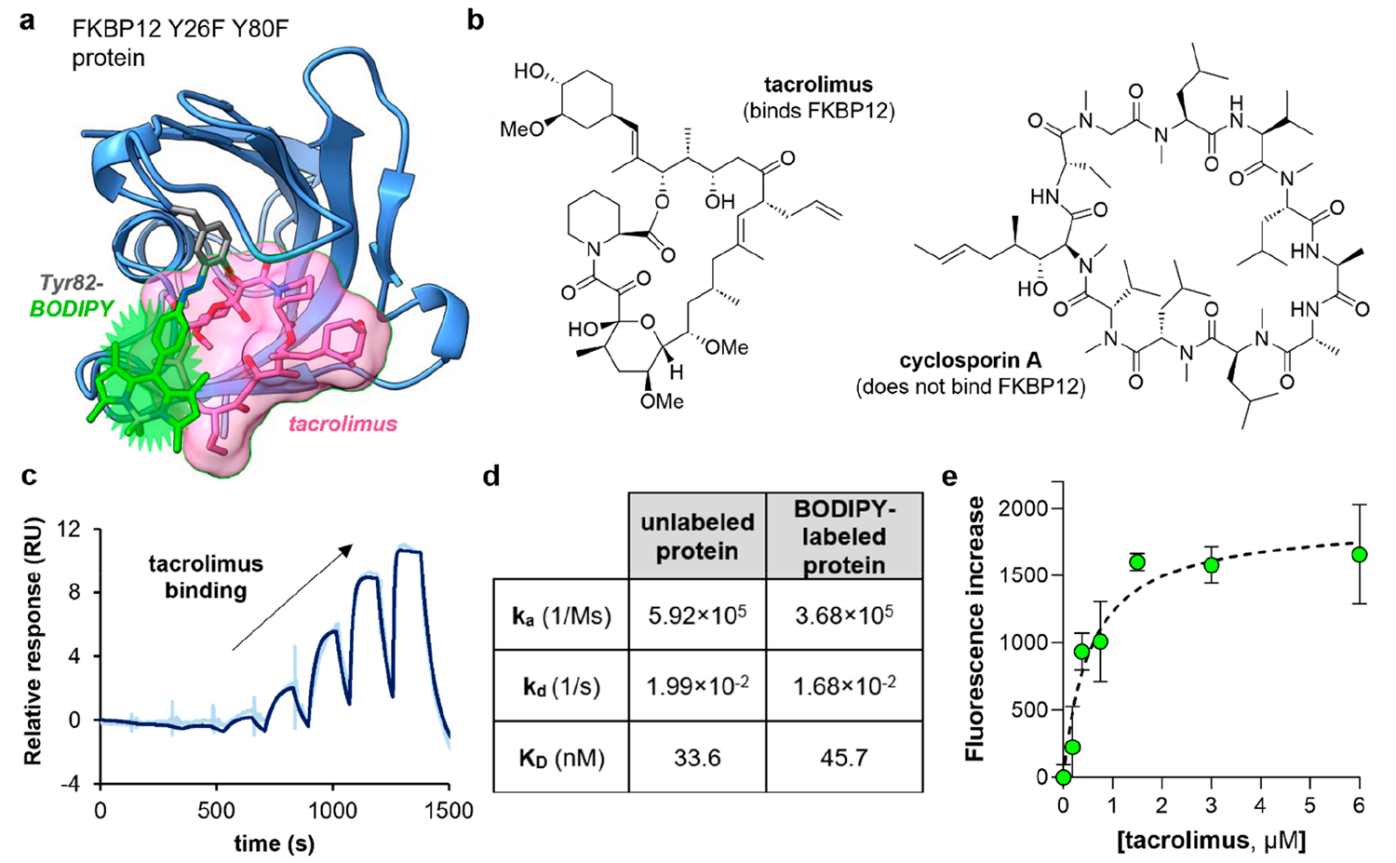
Tyr-specific BODIPY labeling of FKBP12 enables selective fluorescence detection of tacrolimus. (a) Molecular docking illustration displaying the binding of tacrolimus (pink) to FKBP12 Y26F Y80F (blue) labeled with diazonium BODIPY (green). Computational analysis was conducted using DiffDock, and the results were visualized using ChimeraX (PDB code: 1KFJ). (b) Chemical structures of the immunosuppressive drugs tacrolimus and cyclosporin A. (c) Single-cycle kinetic SPR characterization of BODIPY-labeled FKBP12 Y26F Y80F interaction with increasing concentrations of tacrolimus. An 8-point dilution series was performed over a concentration range of 0.2 nM–0.5 μM for tacrolimus. (d) Summary table of the kinetic parameters for SPR binding assays between tacrolimus and unlabeled FKBP12 Y26F Y80F or BODIPY-labeled FKBP12 Y26F Y80F proteins. (e) Fluorescence units’ increase (520 nm) of BODIPY-labeled FKBP12 Y26F Y80F (1 μM) upon binding to increasing concentrations of tacrolimus. Values obtained by subtraction of the emission signal of BODIPY-labeled FKBP12 Y26F Y80F in the presence of tacrolimus vs emission signal in the absence of tacrolimus, presented as means ± SEMs (*n* = 3). The limit of detection (determined as the mean of the blank value plus 3 times the standard deviation of the blank) is equal to 190 nM.

Having optimized the procedure for the Tyr-specific conjugation of BODIPY fluorogens in whole immunophilins, we applied our methodology to protein FKBP12. FKBP12 is a 12 kDa immunophilin that binds to the immunosuppressive drugs tacrolimus and rapamycin, which are macrolides lacking chromophoric groups and cannot be readily detected using conventional optical measurements (Figure 5). First, we analyzed the crystal structure of the complex between human FKBP12 and tacrolimus.^60,61^ The wild-type FKBP12 protein (WT FKBP12) contains three Tyr residues (i.e., Tyr26, Tyr80, and Tyr82), of which the latter is in close proximity to the tacrolimus binding site. We envisaged that the diazonium BODIPY labeling of Tyr82 would (1) retain the ability of FKBP12 to bind tacrolimus with high affinity and (2) incorporate a suitable fluorogen for enhanced turn-on emission upon binding; therefore, we designed a FKBP12 mutant (FKBP12 Y26F Y80F) retaining the native Tyr residue at position 82 and replacing the Tyr26 and Tyr80 residues with Phe (Figure 5). We expressed the FKBP12 Y26F Y80F protein by cloning the DNA fragment corresponding to FKBP12 Y26F Y80F into the pSANG10 plasmid^62^ (Figure S11) and performing protein expression in *E. coli* BL21(pLysS) cells. Cells were lysed by sonication, and the FKBP12 Y26F Y80F protein was purified via the C-terminal 6×His tag (Figure S12). Furthermore, we performed surface plasmon resonance (SPR) binding assays between immobilized FKBP12 Y26F Y80F protein and increasing concentrations of tacrolimus (0.2 nM to 0.5 μM, experimental details in Supporting Information) and observed that the immunophilin retained a high binding affinity for tacrolimus, with *K*_D_ values of around 30 nM (Figure S13).

Using our previously optimized labeling procedure, we then reacted the immunophilin FKBP12 Y26F Y80F with the diazonium BODIPY **3** and obtained DoL values of around 1 (experimental details in the Supporting Information). Importantly, we confirmed by SPR that the BODIPY-labeled FKBP12 Y26F Y80F protein exhibited equally strong binding to tacrolimus (*K*_D_ = 45 nM, Figure 5) and excellent selectivity over other immunosuppressants (i.e., cyclosporin A) that do not bind FKBP12^63^ (Figure S14), thus asserting the nonperturbative character of this labeling approach to generate nativelike fluorogenic proteins. Next, we examined the fluorescence response of BODIPY-labeled FKBP12 Y26F Y80F after incubation with tacrolimus and cyclosporin A as well as other small molecules (e.g., amino acids, lipids, and sugars). Notably, BODIPY-labeled FKBP12 Y26F Y80F showed strong fluorescence emission after incubation with tacrolimus but not in the presence of cyclosporin A and other biological metabolites (Figure S15). These results are in agreement with the in vitro fluorogenicity observed for Tyr-BODIPY (Figure 2), which confirms that the fluorescence emission of labeled immunophilins can be enhanced upon drug binding due to the local increase in hydrophobicity.^64^ Enhanced steric hindrance upon binding may also contribute to the fluorogenic behavior by promoting the restriction of the BODIPY rotation along the phenyl coordinate, leading to a decrease in nonradiative decay, as previously shown in other BODIPY fluorophores.^65^ Other nonradiative transitions such as cis/trans isomeration of the azobenzene moiety may also be contributing factors as reported for other azobenzenes structures.^66,69^ Furthermore, in order to corroborate that Tyr82 was a suitable residue for monitoring the binding of FKPB12 to tacrolimus, we employed the same experimental conditions to label the WT FKBP12 protein as a negative control. In this case, the presence of different Tyr amino acids led to reduced sensitivity for tacrolimus (Figure S16), highlighting the suitability of Tyr82 as an optimal labeling site at the tacrolimus-binding interface.

Finally, we employed the BODIPY-labeled FKBP12 Y26F Y80F in titration assays with tacrolimus and observed a clear dose response reaching saturation levels at equimolar concentrations of protein and a limit of detection of 190 nM (Figure 5). Furthermore, we applied the fluorogenic FKBP12 Y26F Y80F for the detection of tacrolimus in urine samples from renal transplant patients undergoing organ rejection. For these experiments, we analyzed a small collection of biosamples from patients that presented with renal transplant acute kidney injury (AKI) and had been prescribed a twice-daily oral preparation of tacrolimus (Prograf). All patients had been taking tacrolimus for a minimum of 4 weeks, with doses ranging between 1 and 6 mg, and had stable transplant function prior to their AKI. Notably, we observed brighter fluorescence responses of BODIPY-labeled FKBP12 in urine samples from patients when compared to healthy controls (Figure S17). These results indicate the potential application of fluorogenic FKBP12 analogues for the direct detection of immunosuppressive drugs in clinical biosamples and demonstrate that the rational design of site-specific BODIPY-labeled immunophilins is an effective strategy to generate fluorogenic probes for wash-free therapeutic drug monitoring.

## Conclusions

We report the first chemical synthesis of unsubstituted diazonium BODIPY fluorophores as building blocks for the direct derivatization of Tyr amino acids in complex biomolecules. Diazonium BODIPY can be conveniently synthesized by diazotization of the corresponding aniline using commercial reagents in good yields. We demonstrated its utility for the modification of Tyr-containing peptide sequences, such as the self-assembling amyloid peptide KLVYFAE, using conventional SPPS and late-stage protocols, as well as the application to wash-free and real-time microscopy experiments. Furthermore, we optimized the one-step direct coupling of diazonium BODIPY to whole immunophilin proteins under physiological conditions, affording the first fluorogenic analogues of FKBP12. The rational design of a fluorogenic FKBP12 probe where the fluorophore was site-specifically introduced at the tacrolimus-binding interface retained a nativelike selectivity profile and enabled the wash-free detection of nanomolar concentrations of the immunosuppresive drug tacrolimus, including experiments in clinical biosamples from renal transplant patients. The chemical simplicity, modularity, and versatility of this fluorogenic protein labeling strategy have the potential to accelerate the molecular engineering of fluorescent immunophilins for bioanalytical applications.

## Supporting Information Available

Detailed synthetic procedures and full chemical characterization data, methods, supplementary figures, and NMR and IR spectra, and description of Movie S1 (PDF)
Movie S1: time-lapse fluorogenic labeling of peptide aggregates with compound **9** (AVI)
Transparent Peer Review report available (PDF)

## Supplementary Material

oc3c01590_si_001.pdf

oc3c01590_si_002.avi
