# Supplementary material for: Nonperturbative Fluorogenic Labeling of Immunophilins Enables the Wash-free Detection of Immunosuppressants
Source: ACS Cent Sci. 2024 Mar 18;10(5):969–77. doi: 10.1021/acscentsci.3c01590 (PMC11117681; doi:10.1021/acscentsci.3c01590)
Supplement: Supplementary file 1 — oc3c01590_si_001.pdf [file oc3c01590_si_001.pdf]

Electronic Supporting Information

Non-perturbative fluorogenic labeling of immunophilins enables wash-free detection of immunosuppressants

Marco Bertolini,[a,b,¥] Lorena Mendive-Tapia,[a,b,¥] Ouldouz Ghashghaei,[c] Abigail Reese,[a,b] Charles Lochenie,[a,b] Anna M. Schoepf,[c] Miquel Sintes,[c] Karolina Tokarczyk,[d] Zandile Nare,[d] Andrew D. Scott,[d] Stephen R Knight,[e] Advait R. Aithal,[f] Amit Sachdeva,[f] Rodolfo Lavilla,[c,*] Marc Vendrell[a,b,*]

[a] Centre for Inflammation Research, The University of Edinburgh, EH16 4UU Edinburgh, UK. [b] IRR Chemistry Hub, Institute for Regeneration and Repair, The University of Edinburgh, EH16 4UU Edinburgh, UK. [c] Laboratory of Medicinal Chemistry, Faculty Pharmacy and Food Sciences and Institute of Biomedicine UB (IBUB), University of Barcelona, Spain. [d] Concept Life Sciences Ltd, Edinburgh Bioquarter, Edinburgh EH16 4UX, UK. [e] Renal Transplant Unit, Queen Elizabeth Hospital, 1345 Govan Road, Glasgow, G51 4TF. [f] School of Chemistry, University of East Anglia, Norwich, UK.

[¥] These authors contributed equally to this work.

* Corresponding authors' e-mail addresses: rlavilla@ub.edu; marc.vendrell@ed.ac.uk.

Number of pages: 50

Number of figures: 17

Electronic Supporting Information

Table of contents

1. Materials and Methods
2. Supplementary Figures
3. Supplementary Movies
4. NMR and IR Spectra
5. References

1. Materials and methods

Unless otherwise stated, all reactions were carried out under normal atmosphere in dried glassware. All chemicals were purchased from commercial sources and were used as received unless otherwise mentioned. Flash column chromatography was performed on an Isolera Prime Biotage provided with dual UV detection using prepacked normal phase (silica gel) and reverse-phase C18 columns. Thin layer chromatography was performed on precoated Merk silica gel 60 F_{254} plates, which were visualized under UV light at 254 nm and 365 nm. NMR spectra were recorded on 400 MHz or 600 MHz NMR spectrometers. Chemical shifts were reported in ppm (δ) as s (singlet), d (doublet), t (triplet), dd (doublet of doublet), m (multiplet), br s (broad singlet), and were referred to the solvent peak.

Solid-phase peptide synthesis. Amino acids were obtained from Sigma-Merck, Cambridge Bioscience, and Iris Biotech. DIC was obtained from Sigma-Aldrich. TentaGel Rink Amide and Sieber polystyrene resins were obtained from Rapp Polymer GmbH and Novabiochem, respectively. Peptides were manually synthesized in polystyrene syringes fitted with porous polyethylene discs, following standard Fmoc/^{t}Bu SPPS protocols, unless stated otherwise. Fmoc deprotection was carried out using 20% (v/v) piperidine in DMF for 1 min, followed by two 5 min treatments. Subsequently, DMF and DCM washes were performed 5 times each. All syntheses were carried out at r.t. Peptides with fluorescent moieties were consistently protected from light.

HPLC-MS analysis was performed in an Agilent 1260 Infinity at 35°C using a Kinetex XB-C_{18} column (4.6 mm × 50 mm, 5 µm) with H_2O + 0.1% formic acid and ACN + 0.1% formic acid as mobile phases (gradient elution at 1 mL min^{-1}). IR spectra were run on a Perkin-Elmer Spectrum RX I spectrophotometer.

Chemical synthesis

Considering the labile nature of diazonium salts, we aimed at finding conditions in which the diazonium salts **3** could be immediately precipitated in their pure form. Moreover, reducing the amount of the initial solvent using Ar flow before the precipitation slightly improved the isolated yields. However, in some cases it also resulted in impure salts (probably due to the longer retention in the reaction mixture) and was eventually discarded. Figure S1 summarizes the tested conditions to form the diazonium salt **3**. Conditions are modified from reported procedures.[1]

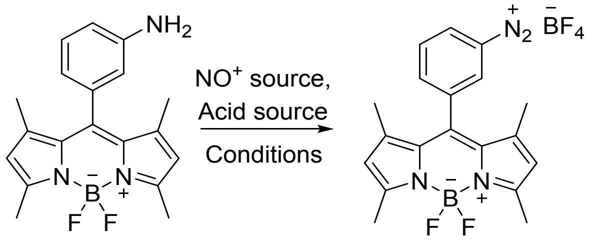

	NO+ source	Acid source	Solvent(s)	Time (temp.)	Yield (%)
**1**	Isoamyl-NO_2 (2 eq)	$BF_3.Et_2O$ (2 eq)	anh. DCM	1 h (0 ºC)	nd
**2**	tBu-NO_2 (1.2 eq)	$BF_3.Et_2O$ (2 eq)	anh. DCM	2 h (0 ºC)	nd
**3**	Isoamyl-NO_2 (4.5 eq)	48 % aq. HBF_4 (6 eq)	EtOH:ACN (2:1)	2 h (-5 ºC to rt)	nd
**4**	Isoamyl-NO_2 (4.5 eq)	48 % aq. HBF_4 (6 eq)	EtOH:DCM (2:1)	3 h (-5 ºC to rt)	39%
**5**	Isoamyl-NO_2 (4.5 eq)	48 % aq. HBF_4 (6 eq)	EtOH:DCM (3:2)	2 h (-5 ºC to rt)	75%[a]
**6**	Isoamyl-NO_2 (4.5 eq)	48 % aq. HBF_4 (6 eq)	EtOH:DCM (3:4)	2 h (-5 ºC to rt)	38%
**7**	Isoamyl-NO_2 (4.5 eq)	48 % aq. HBF_4 (6 eq)	EtOH:DCM (1:1)	2 h (-5 ºC to rt)	84%
**8**	Isoamyl-NO_2 (4.5 eq)	48 % aq. HBF_4 (6 eq)	EtOH:DCM (1:1)	3 h (-20 ºC to rt)	76%

[a] The isolated adduct **3** contained traces of starting material **2**.

Figure S1. Summary of the tested conditions to afford compound **3**. All experiments were run under inert atmosphere and 70-100 mg scale.

5,5-Difluoro-1,3,7,9-tetramethyl-10-(3-nitrophenyl)-5*H*-4λ^4,5λ^4-dipyrrolo[1,2-*c*:2',1'-*f*][1,3,2]diazaborinine (1)

2,4-Dimethylpyrrole (2.77 mL, 27 mmol, 2.4 eq) was added to a solution of 3-nitrobenzoyl chloride (2 g, 10,8 mmol, 1 eq) in dry DCM (33 mL) under Ar. After 72 h of stirring at r.t., Et_3N (9 mL, 64.7 mmol, 6 eq) was added dropwise to the stirring solution. The reaction was cooled to 0 ºC, $BF_3{\cdot}Et_2O$ (10.6 mL, 86.2 mmol, 8 eq) was added and the mixture was stirred for another 48 h. The reaction was then quenched with a saturated solution of Na_2CO_3 and extracted with DCM three times. The combined organic layers were dried with $MgSO_4$, the solvent was removed under reduced pressure and the crude was purified by chromatography (hexane:EtOAc: 0-90%) to obtain **1** as an orange powder (1.31 g, 33%). The ^{1}H NMR data is in accordance with previously reported spectra.[2]

^{1}H NMR (400 MHz, DMSO-*d*$_6$) δ 8.42 (dd, *J* = 2.3, 1.3 Hz, 1H), 8.40 (dd, *J* = 2.3, 1.3 Hz, 1H), 8.28 – 8.26 (m, 1H), 7.91 (dt, *J* = 7.6, 1.4 Hz, 1H), 7.86 (t, *J* = 7.8 Hz, 1H), 6.21 (s, 2H), 2.46 (s, 6H), 1.33 (s, 6H).

3-(5,5-Difluoro-1,3,7,9-tetramethyl-5*H*-4λ^4,5λ^4-dipyrrolo[1,2-*c*:2',1'-*f*][1,3,2]diazaborinin-10-yl)aniline (2)

To a solution of **1** (1.30 g, 3.5 mmol, 1 eq) in DCM (130 mL), was added 130 mg (10%, w/w) of the Pd/C catalyst. The flask was purged with Ar and then with H_2 (1 atm). The mixture was then stirred under the H_2 atmosphere for 48 h, filtered over a Celite pad and washed with DCM (300 mL). The mixture was purified by chromatography (DCM) to obtain **2** as a red solid (881 mg, 74%). The ^{1}H NMR data is in accordance with previously reported spectra.[2]

^{1}H NMR (400 MHz, DMSO-d_6) δ 7.18 (t, *J* = 7.8 Hz, 1H), 6.69 (ddd, *J* = 8.1, 2.2, 0.9 Hz, 1H), 6.48 (t, *J* = 1.8 Hz, 1H), 6.44 – 6.39 (m, 1H), 6.21 (s, 2H), 5.31 (s, 2H), 2.44 (s, 6H), 1.52 (s, 6H).

3-(5,5-Difluoro-1,3,7,9-tetramethyl-5*H*-4λ^4,5λ^4-dipyrrolo[1,2-*c*:2',1'-*f*][1,3,2]diazaborinin-10-yl)benzenediazonium tetrafluoroborate (3)

The aniline **2** (0.080 g, 0.24 mmol, 1 eq) was dissolved in 1 mL of DCM, 1 mL of EtOH and 2-3 drops of $CHCl_3$. Aqueous fluoroboric acid (48% solution, 185 mL, 1.42 mmol, 6 eq) was slowly added while stirring under Ar. The mixture was then cooled to -5 °C. Isoamyl nitrite (143 mL, 1.06 mmol, 4.5 eq) was added dropwise to the reaction mixture and stirring was continued for 1 h at -5 °C and another 1 h at r.t., until the consumption of **2** was confirmed by HPLC-MS. Dilution of the mixture with cold Et_2O (7-10 mL) led to precipitation. The solid was collected by filtration under the flow of Ar and was washed with cold Et_2O to give the pure diazonium salt **3** as a dark red powder (0.087 g, 84%).

^{1}H NMR (400 MHz, CD_3CN) 8.62 (ddd, *J* = 8.5, 2.2, 1.2 Hz, 1H), 8.51 (t, *J* = 2.0 Hz, 1H), 8.32 (dt, *J* = 7.8, 1.5 Hz, 1H), 8.10 (t, *J* = 8.2 Hz, 1H), 6.17 (s, 2H), 2.52 (s, 6H), 1.40 (s, 6H).

^{19}F NMR (376 MHz, CD_3CN) -145.58 (q, *J* = 31.8 Hz), -151.54 (d, *J* = 20.6 Hz).

HRMS (ESI): m/z calculated for $C_{19}H_{18}BF_2N_2^+$ $[M – N_2]^+$: 323.1525, found 323.1529.

Fmoc-Tyr(BODIPY)-OH (4)

In a Schlenk tube under Ar, Fmoc-L-Tyr-OH (0.1 g, 0.25 mmol) was added to a solution of NaOH (1 N, 0.492 mL, 2 eq) and 3 mL H_2O. The tube was immediately transferred to an ice bath. The solution of diazonium salt **3** (0.135 g, 0.31 mmol) in ACN (3 mL) was added dropwise. The reaction mixture was then stirred at 0 °C for 1 h and for up to 2 h at r.t. Once no further evolution was observed, the mixture was diluted with H_2O (10 mL). After confirming the pH was 5-6, the aqueous mixture was extracted with EtOAc (3×15 mL). The combined organic layers were washed with H_2O and brine, dried over $MgSO_4$, and concentrated under reduced pressure. The crude was then purified by reverse-phase flash chromatography (H_2O:ACN, 5% to 100%) to afford the final adduct **4** as an orange fluffy powder (0.060 g, 40%)

^{1}H NMR (400 MHz, DMSO-d_6) δ 10.70 (s, 1H), 8.11 (ddd, J = 8.0, 2.0, 1.1 Hz, 1H), 8.08 (t, J = 1.8 Hz, 1H), 7.83 (d, J = 7.5 Hz, 2H), 7.80 – 7.69 (m, 2H), 7.68 (d, J = 2.2 Hz, 1H), 7.64 – 7.50 (m, 3H), 7.39 – 7.30 (m, 3H), 7.29 – 7.17 (m, 2H), 6.98 (d, J = 8.5 Hz, 1H), 6.19 (d, J = 3.0 Hz, 2H), 4.24 – 4.06 (m, 4H), 3.08 (dd, J = 13.8, 4.4 Hz, 1H), 2.86 (dd, J = 13.8, 10.4 Hz, 1H), 2.47 (s, 6H), 1.38 (s, 6H).

^{13}C NMR (101 MHz, DMSO-d_6) δ 173.2, 155.9, 155.2, 154.1, 152.3, 143.7, 143.6, 142.6, 140.8, 140.6, 140.6, 138.0, 135.1, 135.0, 130.6, 130.5, 130.3, 129.4, 127.5, 126.9, 125.3, 125.1, 121.5, 121.1, 120.5, 120.0, 118.1, 65.6, 55.6, 46.5, 35.6, 14.3.

^{19}F NMR (376 MHz, DMSO-d_6) δ -143.62 (q, J = 30.8 Hz).

HRMS (ESI): m/z calculated for $C_{43}H_{37}BN_5O_5F_2^-$ [M – H]$^-$: 752.2861, found 752.2888.

Peptide 5 (Ac-KLVYFAE-NH_2)

The synthesis was performed on 1.4 g of Tentagel Rink Amide resin (0.18 mmol/g). Fmoc-Lys(Boc)-OH, Fmoc-Tyr(tBu)-OH, Fmoc-Leu-OH, Fmoc-Ala-OH, Fmoc-Val-OH, Fmoc-Phe-OH and Fmoc-Glu(tBu)-OH were used as protected building blocks and their incorporation was carried on a Liberty Blue microwave peptide synthesizer (CEM). For that, DIC and OxymaPure reagents were used for each amide coupling and 20% piperidine in DMF was employed for the removal of Fmoc protecting groups. The peptidyl resins were transferred into a fritted syringe and incubated for 10 min with DIPEA (10 eq.) and Ac_2O (10 eq) in DMF and subsequently washed extensively with DMF and DCM. The dried resin was treated with 5 mL of cleavage cocktail (2.5% H_2O, 2.5% TIPS in TFA) for 1 h before being washed with extra 5 mL. The obtained solution was precipitated by adding cold Et_2O and the resulting precipitate was decanted and dried (×2). Purification was conducted by semi-preparative HPLC using a 5-95% gradient over 15 min, with detection at 254 nm. Pure fractions were lyophilized to afford peptide **5** as a white solid (145 mg, 63% yield).

HPLC: tR: 3.0 min (99% purity).

HRMS (MALDI): m/z calcd. for $C_{45}H_{67}N_9O_{11}$ $[M+H]^+$: 910.5038; found: 910.5138.

Peptide 6 [Ac-KLVY(BODIPY)FAE-NH_2] via SPPS

In a fritted syringe, 21 mgs of Sieber Amide resin (0.57 mmol/g) were swollen 2 mL of DMF for 30 min. Fmoc-Glu(OAll)-OH, Fmoc-Ala-OH, Fmoc-Phe-OH, Fmoc-Val-OH, Fmoc-Leu-OH, Fmoc-Lys(Alloc)-OH and compound para-substituted isomer of compound **4** were used as building blocks. After Fmoc removal, the resin was washed with DMF (×3), DCM (×3) and DMF (×3). Coupling of standard amino acids was carried out using Fmoc-AA-OH (4 eq), COMU (4 eq), OxymaPure (4 eq) and DIPEA (8 eq. in DMF for 1 h at r.t. under constant shaking. For the coupling with Fmoc-Tyr(BODIPY)-OH, the following conditions were used: BODIPY AA (1.2 eq), COMU (1.2 eq), OxymaPure (1.2 eq) and DIPEA (2.4 eq) in DMF. Completion of each coupling step was confirmed using Kaiser Test. After complete elongation of the peptide, the N-terminus acetylation was performed by incubation of peptidyl-resin with Ac_2O (10 eq) and DIPEA (10 eq.) in DMF for 30 min at r.t. After extensive washing with DMF and DCM, on-resin removal of protecting groups was performed incubating the resin with a solution containing dimethyl barbituric acid (3 eq) and $Pd(PPh_3)_4$ (0.1 eq) in DMF. The resin was stirred at r.t. for 30 min and subsequently washed extensively with DMF and DCM. Cleavage of the products from the resin was performed by treatment of resin with 1 mL of 1% TFA in DCM for 10 min at r.t. The solutions were dried, dissolved in DMF and purified by semi-preparative HPLC (5-95%; 15 min; monitoring at 254 nm and 500 nm). The pure fractions were lyophilized to obtain **6** as an orange powder (0.6 mg).

HPLC: tR: 4.6 min (95% purity).

HRMS (MALDI): m/z calcd. for $C_{64}H_{84}BF_2N_{13}O_{11}$ $[M+K]^+$: 1298.6111; found: 1298.6116.

Peptide 6 [Ac-KLVY(BODIPY)FAE-NH_2] via late-stage labeling

Peptide **5** (12.5 mg, 14 µmol, 1 eq) was weighed in a 2 mL tube and dissolved in 200 µL of aqueous NaOH 1 M and 50 µL of dH_2O. The solution was sonicated for 5 min and subsequently cooled down to 0 °C. The para-substituted isomer of the diazonium-BODIPY **3** (7.2 mg, 16 µmol, 1.2 eq) was weighed in a separate tube and dissolved in 150 µL ACN. This solution was transferred dropwise to the vial containing compound **5** and reacted at 0 °C for 30 min under fast stirring. Next, the crude was diluted with 0.6 mL of 1:1 ACN:H_2O+0.1% formic acid and filtered over a short Celite plug. The compound was purified using semi-preparative HPLC (5-95%; 15 min; monitoring at 254 nm and 500 nm). Pure fractions were pooled and lyophilized to afford peptide **6** as an orange powder (2 mg).

HPLC: tR: 4.6 min (95% purity).

HRMS (MALDI): m/z calcd. for $C_{64}H_{84}BF_2N_{13}O_{11}$ $[M+Na]^+$: 1282.6372; found: 1282.7758.

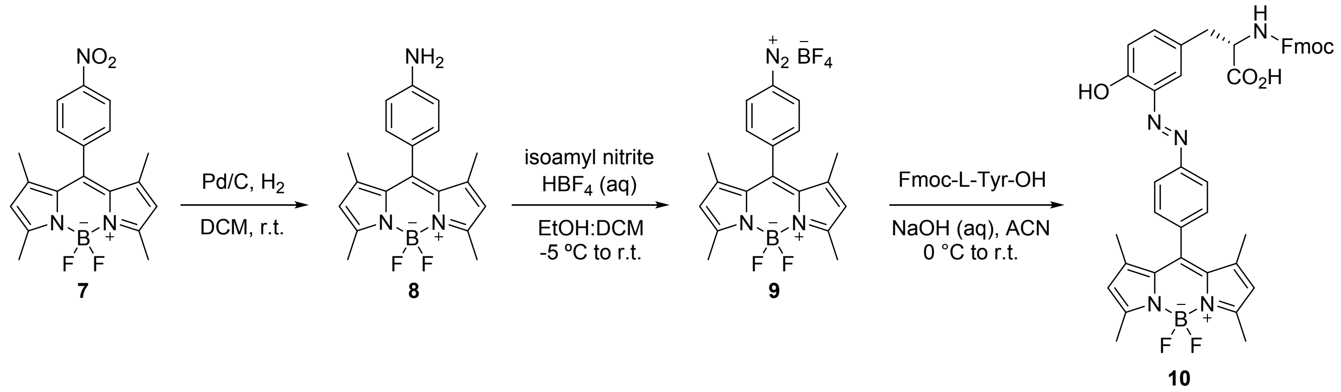

Synthetic scheme S1. Synthesis of *p*-diazonium BODIPY and Tyr-*p*BODIPY adducts.

5,5-Difluoro-1,3,7,9-tetramethyl-10-(4-nitrophenyl)-5*H*-4λ4,5λ4-dipyrrolo[1,2-*c*:2',1'-*f*][1,3,2]diazaborinine (7)

2,4-Dimethylpyrrole (2 mL, 19.4 mmol, 2.4 eq) was added to a solution of 4-nitrobenzoyl chloride (1.5 g, 8.1 mmol, 1 eq) in dry DCM (30 mL) under Ar. After 72 h of stirring at r.t., Et_3N (6.7 mL, 48.5 mmol, 6 eq) was added dropwise to the stirring solution. The reaction was cooled to 0 ºC and $BF_3{\cdot}Et_2O$ (8 mL, 64.7 mmol, 8 eq) was added and the mixture was stirred for another 48 h. The mixture was then quenched with a saturated solution of Na_2CO_3 and extracted with DCM 3 times. The combined organic layers were dried with $MgSO_4$, the solvent was removed under reduced pressure and the crude was purified by chromatography (hexane:DCM: 0-50%) to obtain **7** as an orange powder (0.48 g, 16%). The ^{1}H NMR data is in accordance with previously reported spectra.[3]

^{1}H NMR (500 MHz, DMSO-*d*$_6$) δ 8.40 (d, *J* = 8.7 Hz, 2H), 7.76 (d, *J* = 8.7 Hz, 2H), 6.22 (d, *J* = 1.0 Hz, 2H), 2.47 (s, 6H), 1.34 (s, 6H).

4-(5,5-Difluoro-1,3,7,9-tetramethyl-5*H*-4λ4,5λ4-dipyrrolo[1,2-*c*:2',1'-*f*][1,3,2]diazaborinin-10-yl)aniline (8)

To a solution of **7** (250 mg, 0.66 mmol, 1 eq) in DCM (15 mL), was added 25 mg (10% w/w) of the Pd/C catalyst. The flask was purged with Ar and then with H_2 (1 atm). The mixture was stirred under H_2 atmosphere for 48 h. The crude mixture was filtered over a Celite pad and washed with DCM (100 mL). The solution was concentrated under reduced pressure and purified by chromatography (Hex:AcOEt, 0-80%) to obtain **8** as a red solid (208 mg, 91%). ^{1}H NMR data in accordance with previously reported spectra.[3]

^{1}H NMR (400 MHz, DMSO-d_6) 6.91 (d, *J* = 8.5 Hz, 2H), 6.70 (d, *J* = 8.5 Hz, 2H), 6.14 (s, 2H), 5.43 (s, 2H), 2.43 (s, 7H), 1.50 (s, 6H).

4-(5,5-Difluoro-1,3,7,9-tetramethyl-5*H*-4λ^4,5λ^4-dipyrrolo[1,2-*c*:2',1'-*f*][1,3,2]diazaborinin-10-yl)benzenediazonium tetrafluoroborate (9)

The aniline **8** (0.070 g, 0.2 mmol, 1 eq) was dissolved in 0.5 mL of ACN and 1 mL of EtOH. Aqueous fluoroboric acid (48% solution, 162 mL, 1.24 mmol, 6 eq) was slowly added while stirring under Ar. The mixture was then cooled to -5 °C. Isoamyl nitrite (125 mL, 0.93 mmol, 4.5 eq) was added dropwise to the reaction mixture and stirring was continued for 1 h at -5 °C and another 1 h at r.t., until the consumption of the amino-BODIPY precursor was confirmed by HPLC-MS. Dilution of the mixture with cold Et_2O (7-10 mL) led to precipitation. The solid was collected by filtration under the flow of Ar and was washed with cold Et_2O to give the pure diazonium salt **9** as an orange powder (0.064 g, 71%).

^{1}H NMR (400 MHz, DMSO-d_6) δ 8.85 (d, *J* = 8.7 Hz, 2H), 8.14 (d, *J* = 8.7 Hz, 2H), 6.26 (s, 2H), 2.48 (s, 6H), 1.35 (s, 6H).

^{19}F NMR (376 MHz, DMSO-d_6) δ 143.54 (q, *J* = 32.2 Hz), -148.25 (d, *J* = 21.6 Hz).

HRMS (ESI): m/z calculated for $C_{19}H_{18}BF_2N_2^+$ $[M – N_2]^+$: 323.1525, found 323.1534.

Fmoc-Tyr(*p*BODIPY)-OH (10)

In a Schlenk tube under Ar, Fmoc-L-Tyr-OH (0.070 g, 0.173 mmol) was added to a solution of NaOH (1 N, 0.35 mL, 2 eq.) and 1.5 mL H_2O. The tube was immediately transferred to a 0 °C ice bath. The solution of a para-diazonium salt of compound **9** (0.095 g, 0.22 mmol, 1.2 eq.) in ACN (1.5 mL) was added dropwise. The reaction mixture was stirred at 0 °C for 1 h and for up to 2 h at r.t. Once no further evolution was observed, the mixture was diluted with H_2O (10 mL). After confirming the pH was 5-6, the aqueous mixture was extracted with EtOAc (3×10 mL). The combined organic layers were washed with H_2O and brine, dried over $MgSO_4$, and concentrated under reduced pressure. The crude was then purified by reverse-phase flash chromatography (H_2O:ACN, 5% to 100%) to afford the final adduct **10** as an orange powder (0.053 g, 41%).

^{1}H NMR (400 MHz, DMSO-*d*$_6$) δ 10.88 (s, 1H), 8.13 (d, *J* = 8.3 Hz, 2H), 7.85 (d, *J* = 7.5 Hz, 2H), 7.76 (d, *J* = 8.5 Hz, 1H), 7.70 (d, *J* = 2.2 Hz, 1H), 7.65 – 7.54 (m, 4H), 7.40 – 7.32 (m, 3H), 7.31 – 7.19 (m, 2H), 7.01 (d, *J* = 8.5 Hz, 1H), 6.21 (s, 2H), 4.25 – 4.10 (m, 4H), 3.10 (dd, *J* = 13.9, 4.4 Hz, 1H), 2.89 (dd, *J* = 13.9, 10.5 Hz, 1H), 2.47 (s, 6H), 1.41 (s, 6H).

^{19}F NMR (376 MHz, DMSO-*d*$_6$) δ -143.59 (q, *J* = 32.1 Hz).

^{13}C NMR (101 MHz, DMSO-*d*$_6$) δ 173.2, 155.9, 155.2, 153.8, 151.8, 143.7, 143.7, 140.7, 140.6, 138.2, 136.5, 135.0, 130.5, 129.5, 129.2, 127.6, 127.0, 125.3, 125.2, 123.5, 122.2, 121.6, 120.1, 118.1, 65.6, 55.6, 46.6, 35.6, 14.3, 14.2.

HRMS (ESI): m/z calculated for $C_{43}H_{37}BN_5O_5F_2^-$ [M – H]$^-$: 752.2861, found 752.2843.

Protein labeling and characterization. PPIA and FKBP12 proteins were diluted in ice-cold carbonate buffer (0.1 M, pH=9) at a final concentration of 5 µM (1 eq). Diazonium-BODIPYs were dissolved in DMSO at a final concentration of 0.5 mM. A volume equal to 5 eq of diazonium BODIPY was transferred to the protein solution and the mixture was incubated under constant stirring at 4 °C for 1 h. For the PPIA protein, buffer exchange and removal of excess dye were performed using Amicon Ultra 0.5mL centrifugal filters (MWCO 3 kDa). The conjugation mixture was diluted with PBS to reach a final volume of 500 µL, loaded onto the ultrafiltration unit and centrifuged at 21,000 rcf for 30 min at 4 °C. This procedure was repeated 3 times to ensure complete removal of excess dye. For the FKBP12 proteins, buffer exchange and removal of excess dye were performed using Zeba Spin Desalting Column (0.5 mL, MWCO 7 kDa). Purifications were performed according to the manufacturer's protocol by equilibrating the column 3 times with PBS. Following purification, the concentrations of the labeled proteins concentration were determined using the Coomassie Plus Bradford Assay Kit in accordance with the manufacturer's guidelines. The BODIPY absorbance values of the conjugates were recorded at 500 nm on a NanoDrop One spectrophotometer and the degree of labeling (DoL) was calculated by dividing the concentration obtained from the absorbance measurement by the concentrations determined using the Bradford Assay. SDS-PAGE were performed with NuPAGE™ 4-12% Bis-Tris Gels. Precast gels were loaded with 3 µg protein (2× non-reducing Laemmli SDS sample buffer) to run the gel for 45 min at 200 V in NuPAGE™ MOPS SDS Running Buffer. For Coomassie-based protein visualization, the gel was subjected to an overnight incubation in Coomassie staining solution, and then destained in miliQ H_2O. In-gel fluorescence was acquired on a FujiFilm FLA-5100 Fluorescent Image Analyzer at ~500 nm.

Construction of pSANG10_(-PelB)FKBP12 Y26F Y80F. Our groups have used pSANG10-7D12 plasmid for the periplasmic expression of the nanobody 7D12.[4] This plasmid was digested with NdeI and HindIII to remove the coding DNA sequence (CDS) for PelB leader

peptide and 7D12 fragment. After digestion, the reaction mixture was run on a 1% agarose gel. The band corresponding to pSANG10 backbone was excised and extracted using QIAquick Gel extraction kit. The codon optimized CDS for FKBP12 Y26F Y80F with a C-terminal 6xHis and Gibson overhangs was subsequently cloned into the pSANG10 backbone using Gibson cloning. After cloning, the sequence of pSANG10_(-PelB)FKBP12 Y26F Y80F was confirmed by Sanger sequencing.

Expression of FKBP12 Y26F Y80F protein. For expression of FKBP12 Y26F Y80F, the pSANG10_(-PelB)FKBP12 Y26F Y80F plasmid was transformed into 50 mL BL21(DE3)pLysS chemically competent cells. After transformation, cells were recovered in 0.5 mL SOB medium for 1 h at 37 °C. 50 µL of recovered cells were used to inoculate 10 mL of 2×TY-GK media (2xTY media with 2% glucose, 50 µg mL^{-1} kanamycin) and incubated overnight (37 °C, 220 rpm, 16 h). Next day, this culture was used to subculture 500 mL 2×TY-GK, such that OD_{600}= 0.1. This was then incubated until OD_{600} reached 0.4-0.6 (37 °C, 220 rpm, ~2 h). IPTG (1 mM final concentration) was added to induce the expression of FKBP12 Y26F Y80F and the culture was incubated overnight (30 °C, 160 rpm, 16 h). Next day, cells were pelleted (3, 200g, 4 °C, 10 min). The supernatant was discarded, and the cells were resuspended in 10 mL PBS supplemented with one protease inhibitor cocktail tablet, 1 mg mL^{-1} lysozyme, 1 mg mL^{-1} DNase I (Merck). The cells were then lysed by sonicating the cell suspension thrice while on ice. After sonication, the cell suspension was centrifuged (14,000 *g*, 4 °C, 10 min) and the supernatant was collected in a separate tube. For purification of FKBP12 Y26F Y80F, 1 mL of Ni-NTA resin was added to the supernatant and mixed gently on a rocker (4 °C, 1 h). This suspension was then transferred into a gravity-flow column and washed with 20 mL PBS. Resin was then washed twice with 20 mL Ni-NTA wash buffer (PBS containing 20 mM imidazole). To elute the bound FKBP12 Y26F Y80F, 500 µL Ni-NTA elution buffer (PBS containing 200 mM imidazole) was added and incubated for 15 min at r.t. These steps were repeated 8 times. Elution fractions were

pooled, diluted to 40 mL PBS, and the purification step was repeated. The collected fractions were again pooled and diluted to 40 mL using PBS. The final round of purification was performed using AKTA prime plus. The sample was loaded on to HiTrap Ni^{2+} chelating column (1 mL), previously washed with buffer B (PBS containing 300 mM imidazole) and then equilibrated with buffer A (PBS). The bound proteins were eluted (0.5 mL min^{-1}) using a linear gradient from 0 to 100% (v/v) buffer B. 0.5 mL fractions were collected, fractions containing the FKBP12-Y26F Y80F protein were pooled and analyzed by SDS PAGE. For SDS-PAGE analysis, samples were heated at 95 °C for 15 min, then centrifuged (13,000 g, 15 min, 4 °C) and loaded on a 4-12% Bis-Tris gel. The gel was then stained with Coomassie Blue, and the identity of the protein was further confirmed by electrospray ionization mass spectrometry coupled with liquid chromatography.

Labeling of FKBP12 Y26F Y80F protein. FKBP12 Y26F Y80F protein was diluted to a concentration of 5 µM in ice-cold carbonate buffer (pH 9, 100 mM). Subsequently, 5 equivalents of a diazonium BODIPY solution in DMSO were added to the protein solution. The reaction mixture was kept at 4 °C in the dark with constant shaking for 1 h. The resulting reaction crude was subjected to purification using Zeba Spin desalting columns (0.5 mL, MWCO 7 kDa) following the manufacturer's protocol. The concentration of the BODIPY-labeled FKBP12 Y26F Y80F was determined using the Coomassie Plus Bradford Assay Kit. The absorbance values at 280 nm and 500 nm of BODIPY-labeled proteins were measured using a NanoDrop One spectrophotometer and correlated to the calibration curve of diazonium BODIPY in the presence of 0.1% bovine serum albumin (BSA) to calculate the DoL.

Surface Plasmon Resonance (SPR) binding assays. The equilibrium dissociation constant (K_D) and the association and dissociation rate constants (k_a and k_d) were assessed using a Biacore 8K SPR instrument (Cytiva) and single-cycle kinetic (SCK) analysis. A combined His-tag capture/amine coupling approach was used to generate a stable surface on an NTA sensor chip (Cytiva). Briefly, the NTA groups were first chelated with nickel ions (0.5 M $NiCl_2$), activated with 0.2 M 1-ethyl-3-(3-dimethylaminopropyl) carbodiimide (EDC) and 50 mM N-hydroxysuccinimide (NHS) before a 200 s injection of FKBP12 proteins and subsequent covalent immobilization. The running buffer was 20 mM phosphate buffer containing 2.7 mM KCl and 0.05 % (v/v) Tween 20 (PBS-P+, pH 7.4). The wild-type FKBP12, FKBP12 Y26F Y80F, and BODIPY-labeled FKBP12 Y26F Y80F proteins were diluted to 1, 2, and 7 $\mu g\ mL^{-1}$ in the running buffer to achieve immobilization levels of 1057, 1091, and 1029 RU, respectively. After successful immobilisation, Ni^{2+} ions were removed with an injection of 0.35 M EDTA (Cytiva, Sweden). Following this, SCK experiments were run using PBS-P+ supplemented with 1 % DMSO (v/v). Cyclosporin A and tacrolimus were flowed over immobilized proteins for 120 s at a rate of 30 $\mu L\ min^{-1}$. An 8-point dilution series was performed over a concentration range of 0.2 nM–0.5 μM for tacrolimus and 3 nM–10 μM for cyclosporin A. All steps were carried out at 25 °C. The sensorgrams were referenced (activated and deactivated surface) and blank-subtracted (blank cycle with zero analyte concentration). The data was evaluated using a 1:1 kinetic binding model by the Biacore Insight Evaluation software.

Clinical biosample assays. Ethical approval was gained from the East of Scotland Research Ethics Service (Reference: GN22RE301). Urine samples (5 mL) from patients with transplant acute kidney injury and healthy donors underwent rapid freezing in liquid nitrogen followed by overnight lyophilization. After reconstitution in 1 mL of sterile dH_2O and filtration through 0.45 μm filters, a solution of BODIPY-labeled FKBP12 Y26F Y80F (1 μM) was added, and 25 μL of the solutions were dispensed into a 384-well plate. Fluorescence readings were recorded for 12

h using a Varioskan Lux spectrophotometer, with an excitation wavelength of 495 nm and an emission wavelength of 515 nm. The measured fluorescence ratios between t = 0 and t = 12 h were obtained for each sample.

2. Supplementary figures

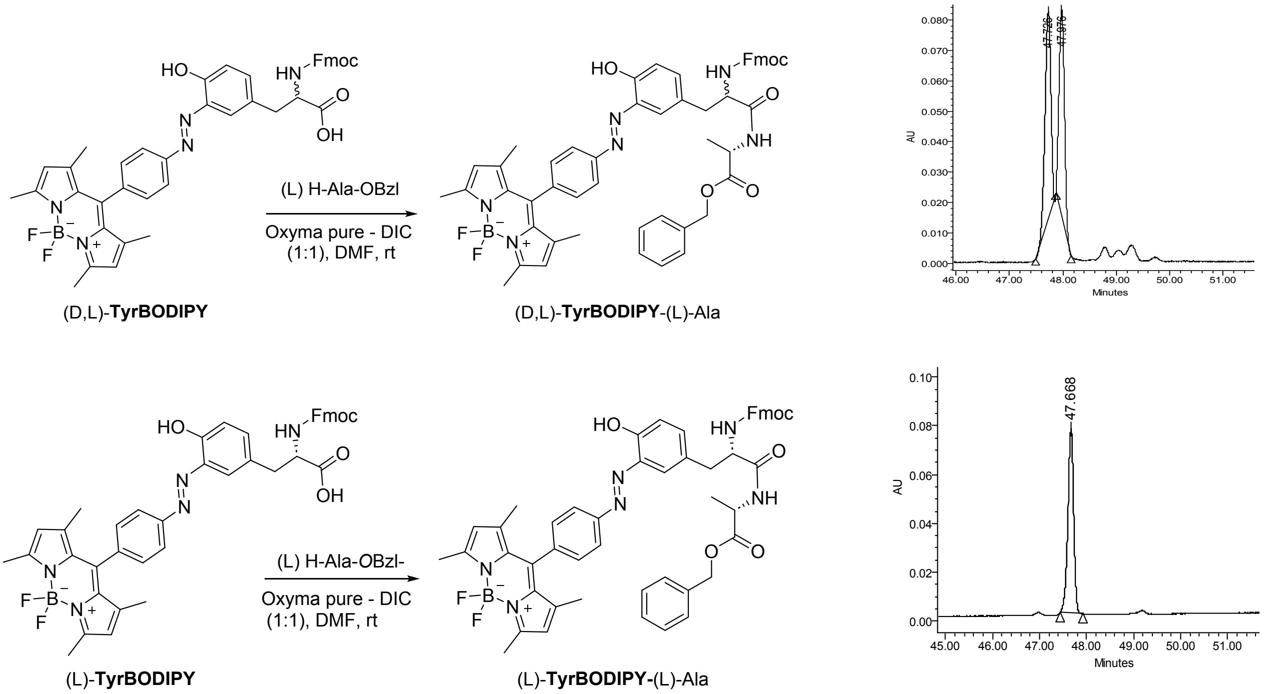

Figure S2. Enantiopurity analysis of Fmoc-Tyr(BODIPY)-OH. Top) the racemic mixture (D,L)-Fmoc-Tyr(BODIPY)-OH was coupled to H-L-Ala-OBzl to form the two diastereomers DL and LL. Bottom) the enantiopure (L)-Fmoc-Tyr(BODIPY)-OH was coupled to H-L-Ala-OBzl. The top chromatogram showed two peaks whereas the bottom chromatogram showed a single peak, indicating that the coupling reaction under the conditions described does not result in detectable racemization of the amino acid.

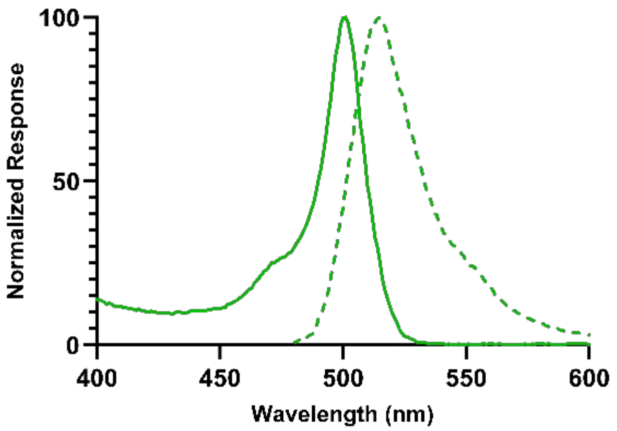

Figure S3. Representative absorbance (solid) and emission (dashed) spectra of compound **4** in EtOH. Spectroscopic data were recorded on a Synergy HT spectrophotometer. Stock solutions were freshly prepared in DMSO (2.5 mM) and were then used to prepare 25 μM solutions of compound **4** in EtOH. Spectra were recorded at r.t. and are represented as means from 3 independent experiments.

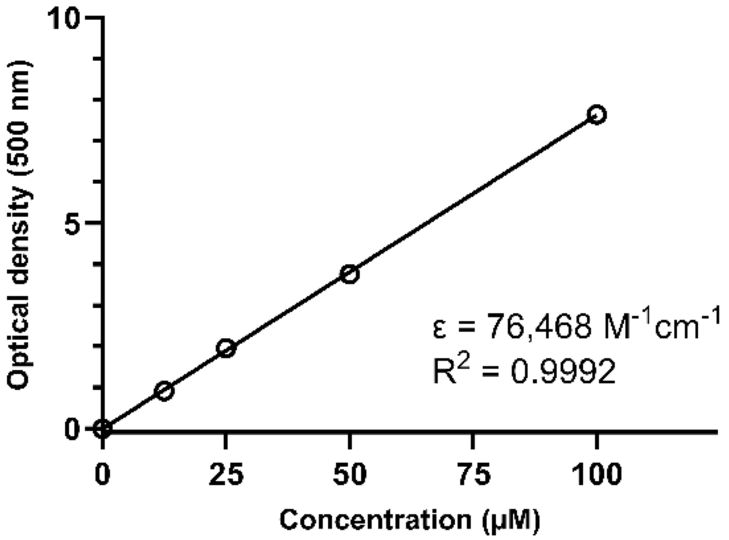

Figure S4. Extinction coefficients of compound **4** in EtOH. Absorption values were measured on a NanoDrop One spectrophotometer. Values presented as means±SD (n=5). λ_{abs}: 500 nm.

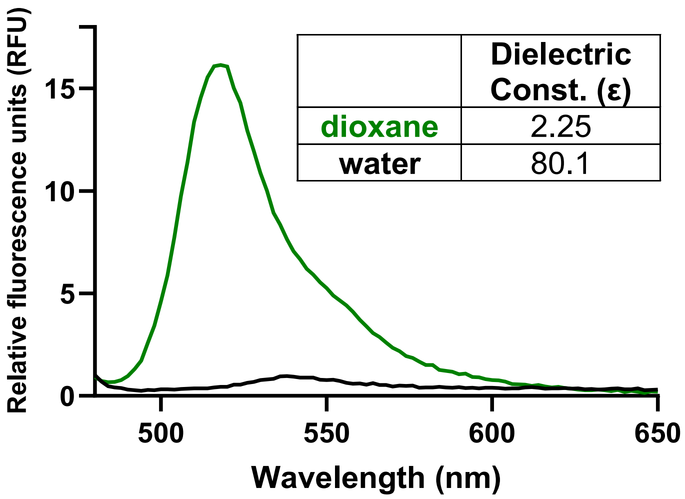

Figure S5. Representative emission spectra of compound **4** (50 μM) in water (black) and dioxane (green). λ_{exc}: 450 nm (n=3).

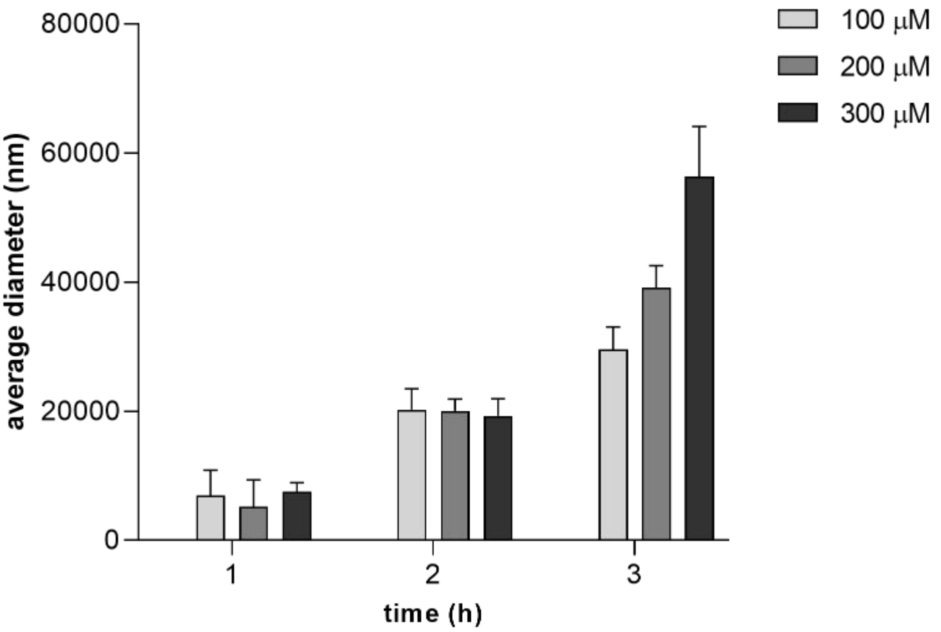

Figure S6. Dynamic light scattering measurement of peptide **5** aggregates. The aggregation kinetics of peptide **5** at the indicated concentrations and time points were measured using a NanoBrook 90Plus instrument (Brookhaven Instruments). Values presented as means±SD from 3 independent experiments.

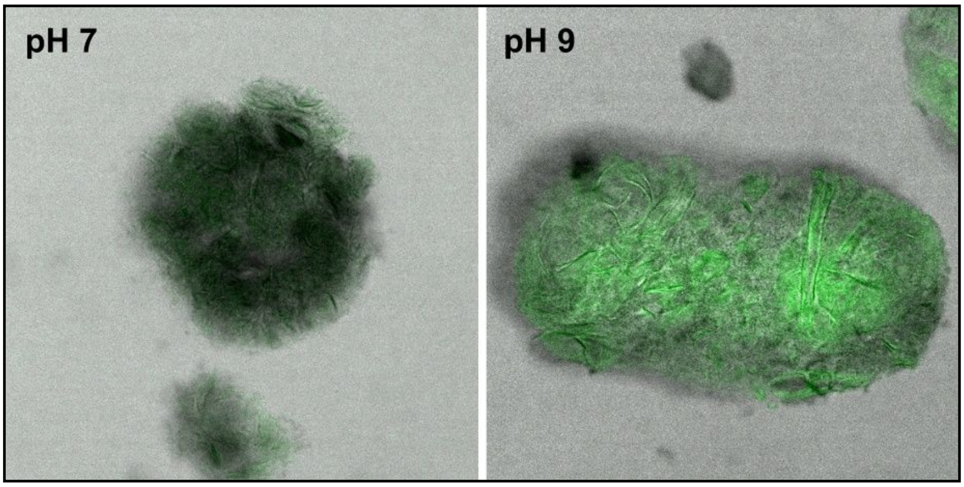

Figure S7. Fluorescence imaging (merged of brightfield and fluorescence images) of peptide **5** aggregates. Self-aggregates of peptide **5** (100 μM) were prepared in PBS (pH 7) or in carbonate buffer (pH 9) and transferred to a μ-Slide 8-well chamber and incubated with diazonium BODIPY (10 μM) at r.t. followed by image acquisition using a Leica SP8 microscope (λ_{exc}: 488 nm).

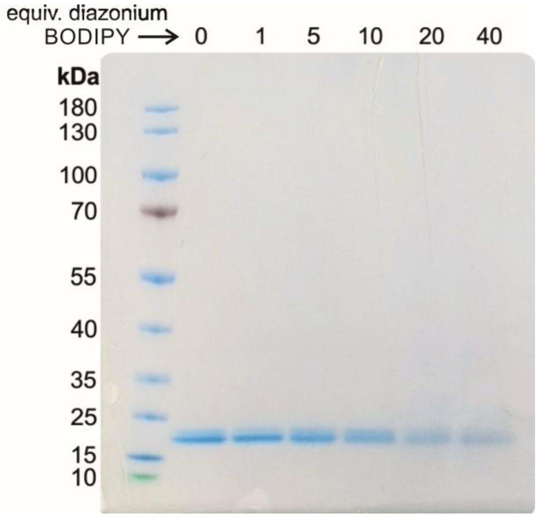

Figure S8. Coomassie Blue-stained SDS-PAGE analysis for the different labeling reactions of PPIA with variable equivalents of diazonium BODIPY (Mw (PPIA): 18,012 Da).

MASCOT Search Results

Protein View: sp|P62937|PPIA_HUMAN

Peptidyl-prolyl cis-trans isomerase A OS=Homo sapiens GN=PPIA PE=1 SV=2

Database: Hsapiens_uniprot
Score: 6396
Nominal mass (M_r): 18229
Calculated pI: 7.68

Sequence similarity is available as an NCBI BLAST search of sp|P62937|PPIA_HUMAN against nr.

Search parameters

MS data file: 221117_o2_07_Marco_test_02.mgf
Enzyme: Trypsin/P: cuts C-term side of KR.
Fixed modifications: Carbamidomethyl (C)
Variable modifications: Oxidation (M), Azo-BODIPY-dye (Y)

Protein sequence coverage: 98%

Matched peptides shown in ***bold red***.

```
  1 MVNPTVFFDI AVDGEPLGRV SFELFADKVP KTAENFRALS TGEKGFGYKG
 51 SCFHRIIPGF MCQGGDFTRH NGTGGKSIYG EKFEDENFIL KHTGPGILSM
101 ANAGPNTNGS QFFICTAKTE WLDGKHVVFG KVKEGMNIVE AMERFGSRNG
151 KTSKKITIAD CGQLE
```

Tyr-BODIPY detected on the peptide **GFRYKGSCFHR**

**Protein Accession No.**	sp|Q9Y536|PAL4A_HUMAN
**Peptide Sequence**	**GFRYKGSCFHR**
**Peptide missed cleavages**	2
**Peptide modification**	Azo-BODIPY dye (Y)
**Expected peptide mass**	**1764.8301**
**Detected peptide mass**	**1764.8301**

Peptide Fragment Chain*	Fragment size
R	175.119
RH	312.1779
RHF	459.2463
RHFC	619.2769
RHFCS	706.309
RHFCSG	763.3304
RHFCSGK	891.4254
**RHFCSGKY**	1405.648
RHFCSGKYR	1561.7491
RHFCSGKYRF	1708.8175
RHFCSGKYRFG	1764.8301

* Identified fragments highlighted in blue.

MASCOT Search Results

Protein View: sp|P62937|PPIA_HUMAN

Peptidyl-prolyl cis-trans isomerase A OS=Homo sapiens GN=PPIA PE=1 SV=2

Database: Hsapiens_uniprot
Score: 6194
Nominal mass (M_r): 18229
Calculated pI: 7.68

Sequence similarity is available as an NCBI BLAST search of sp|P62937|PPIA_HUMAN against nr.

Search parameters

MS data file: 230111_o2_05_marco_hu_PPIA_03.mgf
Enzyme: Trypsin/P: cuts C-term side of KR.
Fixed modifications: Carbamidomethyl (C)
Variable modifications: Oxidation (M), Azo-BODIPY-dye (Y)

Protein sequence coverage: 98%

Matched peptides shown in ***bold red***.

```
  1 MVNPTVFFDI AVDGEPLGRV SFELFADKVP KTAENFRALS TGEKGFGYKG
 51 SCFHRIIPGF MCQGGDFTRH NGTGGKSIYG EKFEDENFIL KHTGPGILSM
101 ANAGPNTNGS QFFICTAKTE WLDGKHVVFG KVKEGMNIVE AMERFGSRNG
151 KTSKKITIAD CGQLE
```

Tyr-BODIPY detected on the peptide **HNGTGGKSIYGEKFEDENFILK**

**Protein Accession No.**	sp|P62937|PPIA_HUMAN
**Peptide Sequence**	**HNGTGGKSIYGEKFEDENFILK**
**Peptide missed cleavages**	2
**Peptide modification**	Azo-BODIPY dye (Y)
**Expected peptide mass**	**2833.3519**
**Detected peptide mass**	**2833.3721**

Peptide Fragment Chain*	Fragment size
K	147.1128
LK	260.1969
ILK	373.2809
FILK	520.3493
NFILK	634.3923
ENFILK	763.4349
DENFILK	878.4618
EDENFILK	1007.5044
FEDENFILK	1154.5728
KFEDENFILK	1282.6678
EKFEDENFILK	1411.7104
GEKFEDENFILK	1468.7318
**YGEKFEDENFILK**	**1982.9544**
IYGEKFEDENFILK	2096.0385
SIYGEKFEDENFILK	2183.0705
KSIYGEKFEDENFILK	2311.1655
GKSIYGEKFEDENFILK	2368.1869
GGKSIYGEKFEDENFILK	2425.2084
TGGKSIYGEKFEDENFILK	2526.2561
GTGGKSIYGEKFEDENFILK	2583.2775
NGTGGKSIYGEKFEDENFILK	2697.3205
HNGTGGKSIYGEKFEDENFILK	2833.3721

* Identified fragments highlighted in blue.

Figure S9. Mass spectrometry analysis of BODIPY-labeled PPIA after trypsin digestion. Diazonium BODIPY conjugation was observed in the two different Tyr residues found in the PPIA protein.

Protein	Modification (Residue)	Experimental peptide mass	Calculated peptide mass	Peptide sequence
**PPIA**	2 Oxidation (M)	1309.5602	1309.5642	EGMNIVEAMER
**PPIA**	2 Oxidation (M)	1536.7344	1536.7276	VKEGMNIVEAMER
**PPIA**	Oxidation (M)	1293.5722	1293.5693	EGMNIVEAMER
**PPIA**	Oxidation (M)	1520.7211	1520.7327	VKEGMNIVEAMER
**PPIA**	Oxidation (M)	1613.7346	1613.7331	IIPGFMCQGGDFTR
**PPIA**	Oxidation (M)	1740.8016	1740.7923	EGMNIVEAMERFGSR
**PPIA**	Oxidation (M)	1967.9659	1967.9557	VKEGMNIVEAMERFGSR
**PPIA**	Oxidation (M)	2092.0198	2092.0300	MVNPTVFFDIAVDGEPLGR
**PPIA**	Oxidation (M)	2188.1273	2188.1133	HVVFGKVKEGMNIVEAMER
**PPIA**	Oxidation (M)	2265.0561	2265.0419	IIPGFMCQGGDFTRHNGTGGK
**PPIA**	Oxidation (M)	2806.3416	2806.3167	HTGPGILSMANAGPNTNGSQF FICTAK
**PPIA**	Oxidation (M)	4303.1163	4303.0943	HTGPGILSMANAGPNTNGSQF FICTAKTEWLDGKHVVFGK

Figure S10. Proteomic analysis for BODIPY-labeled PPIA. All peptide fragments were examined for the potential labeling of Phe and Trp residues. The only detected modifications were oxidation products in Met residues formed during the digestion process.

TAACTTTAAGAAGGAGATATACATATGGGGGTGCAAGTCGAGACGATTTCGCCCGGAGACGGGCGCACT

TTCCCAAAGCGCGGTCAGACTTGTGTAGTACACTTCACAGGAATGCTGGAAGATGGCAAAAAATTCGAT

AGTTCGCGTGACCGCAATAAGCCTTTCAAGTTTATGCTGGGGAAGCAAGAGGTCATTCGCGGCTGGGAG

GAAGGTGTCGCACAAATGTCTGTGGGTCAACGCGCCAAATTAACTATCTCTCCGGATTTCGCCTACGGC

GCAACGGGCCATCCAGGAATCATCCCTCCCCACGCCACGTTAGTGTTCGACGTGGAATTGTTGAAGTTA

GAGCACCACCATCACCACCACTGATAAAAGCTTTAATAAGTCGAGCACCACCACC

Figure S11. Sequence of gene block inserted into pSANG10 plasmid for generation of pSANG10_(-PelB)FKBP12 Y26F Y80F plasmid. Red: Gibson overhangs; underlined: restriction sites used for cloning; green: DNA sequence for expression of FKBP12 Y26F Y80F; blue: 6×His tag.

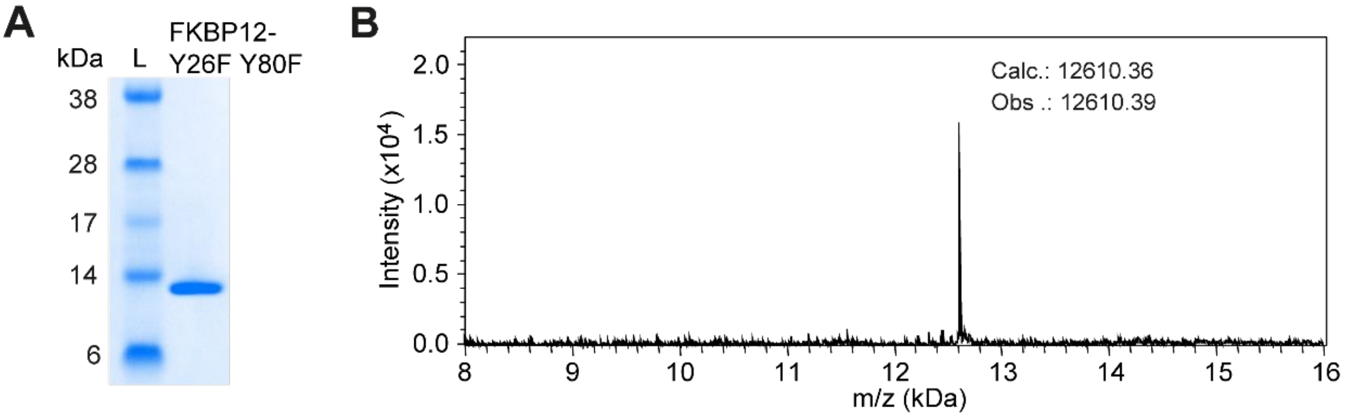

Figure S12. Expression of the FKBP12 Y26F Y80F protein using the pSANG10_(-PelB)FKBP12 Y26F Y80F plasmid. A) Coomassie-stained gel image showing expression and purity of FKBP12 Y26F Y80F after purification. L: Invitrogen SeeBlue Plus2 Prestained Protein Standard. B) ESI-MS of the FKBP12 Y26F Y80F protein to confirm identity (Note: the calculated molecular weight does not include the first methionine).

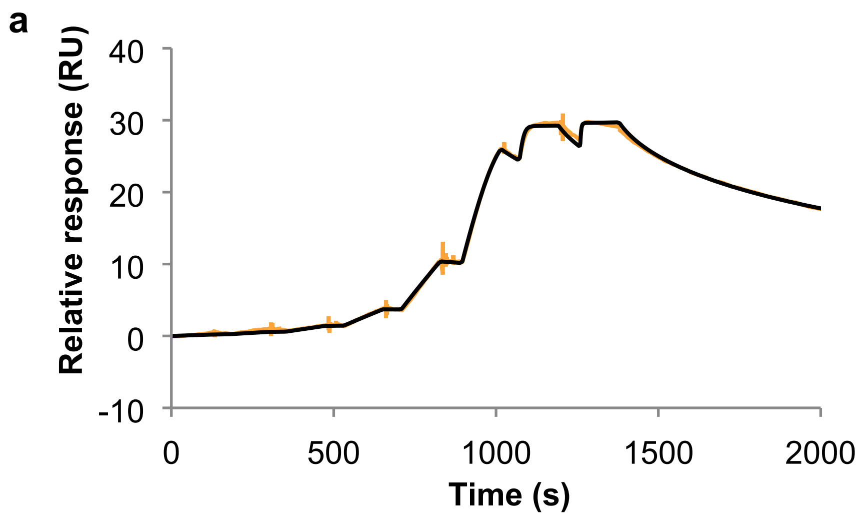

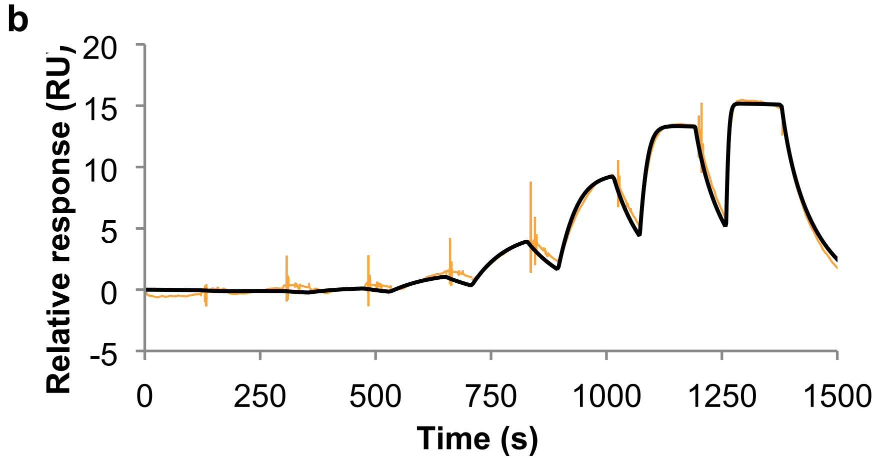

Ligand	Immunosuppressant	k_a (1/Ms)	k_d (1/s)	K_D (nM)
WT FKBP12	cyclosporin A		no binding	
	tacrolimus	1.04×10^{6}	2.48×10^{-3}	2.40
FKBP12 Y26F Y80F	cyclosporin A		no binding	
	tacrolimus	5.92×10^{5}	1.99×10^{-2}	33.6

Figure S13. Single-cycle kinetic SPR characterization of the binding between tacrolimus (0.2 nM-0.5 μM) and immobilized WT FKBP12 (a) or FKBP12 Y26F Y80F (b). Summary table with kinetic parameters for the interactions between immunosuppressants and WT FKBP12 and FKBP12 Y26F Y80F proteins. For cyclosporin A, no measurable binding to either protein was detected at concentrations of drug up to 10 μM.

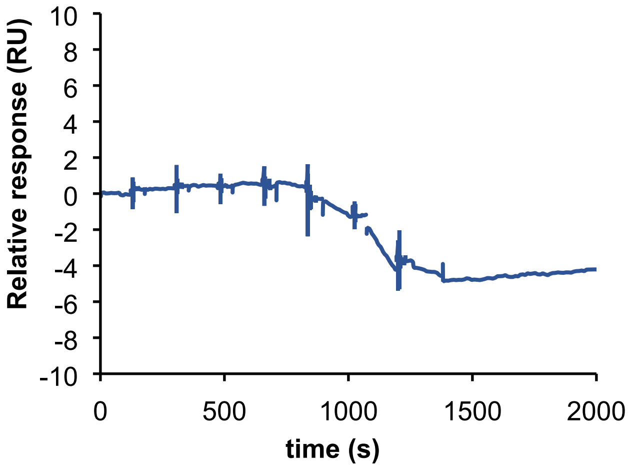

Figure S14. Single-cycle kinetic SPR characterization of BODIPY-labeled FKBP12 Y26F Y80F interaction with increasing concentrations of cyclosporin A as a negative control. An 8-point dilution series was performed over a concentration range of 3 nM-10 μM for cyclosporin A.

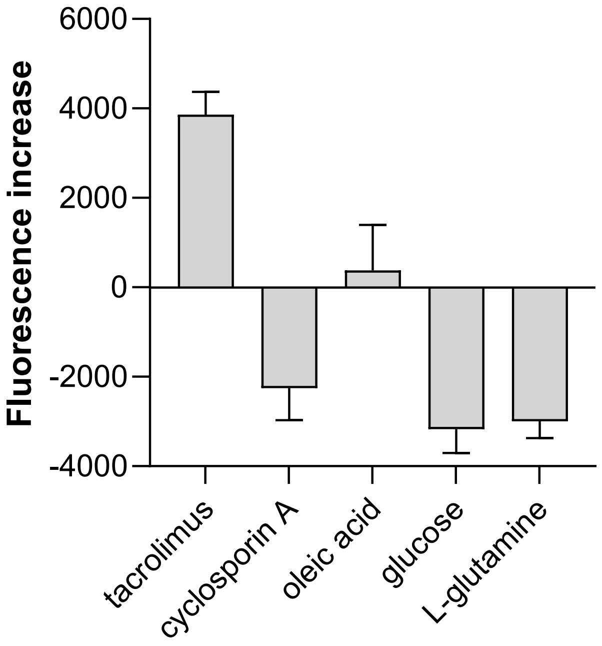

Figure S15. Wash-free fluorescence emission (520 nm) of the BODIPY-labeled FKBP12 Y26F Y80F (1 μM) after incubation with immunosupressive drugs and other biomolecules (all at 5 μM). Values obtained by substraction of the emission signal of BODIPY-labeled FKBP12 Y26F Y80F in the presence of biomolecule vs emission signal in the absence of biomolecule, and presented as means±SEM (n=3).

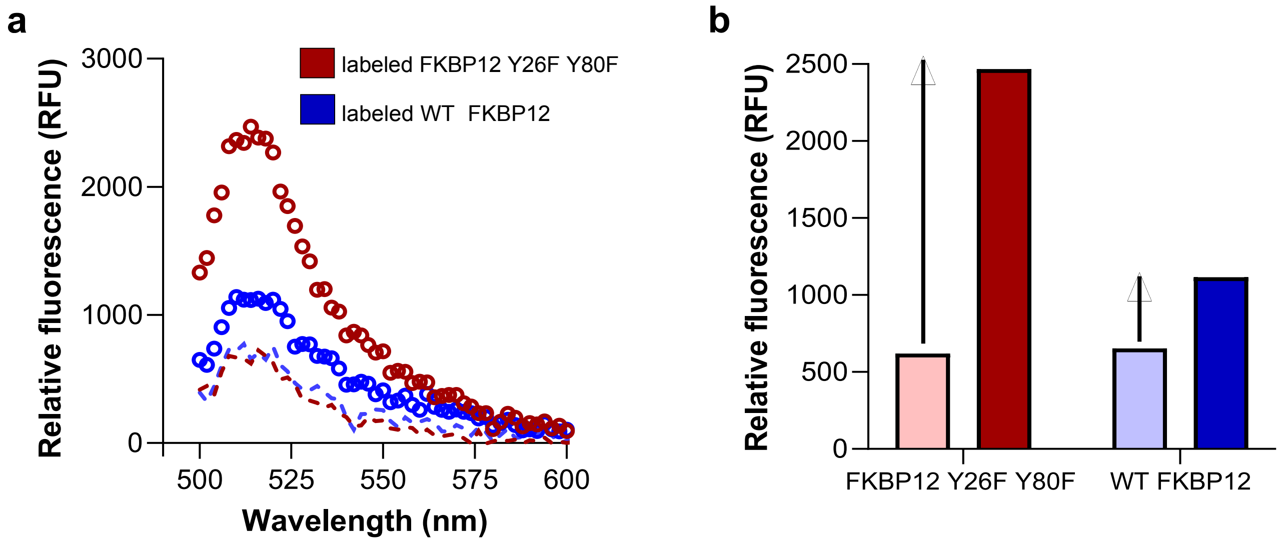

Figure S16. a) Representative emission spectra of BODIPY-labeled proteins FKBP12 Y26F Y80F (red, 1 µM) and WT FKBP12 (blue, 1 µM) in the absense (dashed lines) or presence (circles) of tacrolimus (5 µM). λ_{exc}: 450 nm. Values are represented as means±SEM from two independent experiments. b) Fluorescence intensity of BODIPY-labeled proteins FKBP12 Y26F Y80 (red, 1 µM) and WT FKBP12 (blue, 1 µM) in the absence of tacrolimus (5 µM) (light-colored bars) or presence of tacrolimus (5 µM) (dark-colored bars). λ_{exc}: 450 nm. Values are represented as means from two independent experiments.

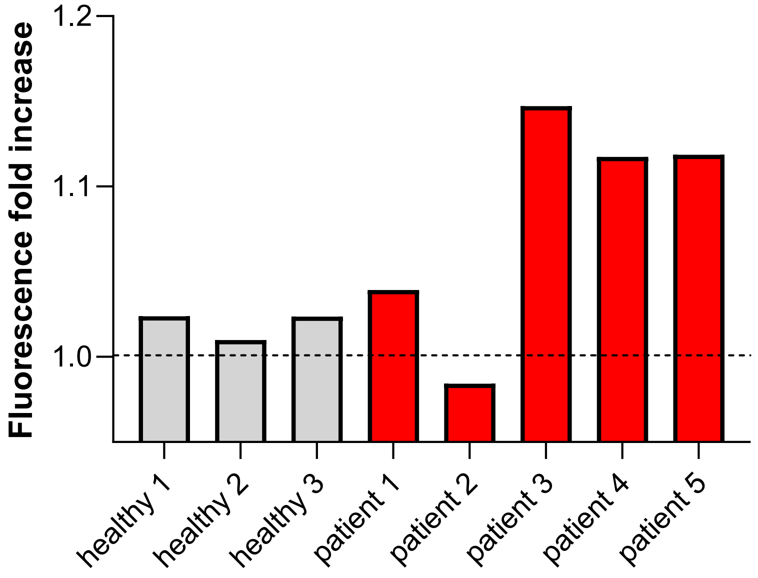

Figure S17. Fluorescence fold increases (520 nm) of BODIPY-labeled FKBP12 Y26F Y80F (1 μM) upon incubation with urine samples from healthy controls and tacrolimus-treated patients with transplant acute kidney injury. Values were obtained by referring the fluorescence emission signals of BODIPY-labeled FKBP12 Y26F Y80F after 12 h incubation vs the fluorescence emission signals at time 0.

4. Supplementary Movies

Movie S1. Time-lapse fluorogenic labeling of peptide aggregates with compound **9**. Peptide **5** was incubated in carbonate buffer pH 9 (100 μM) with compound **9** (5 μM). Movies were recorded for 10 min and compressed (jpeg) at 10 fps.

5. NMR and IR Spectra

Compound **1** (DMSO-d_6)

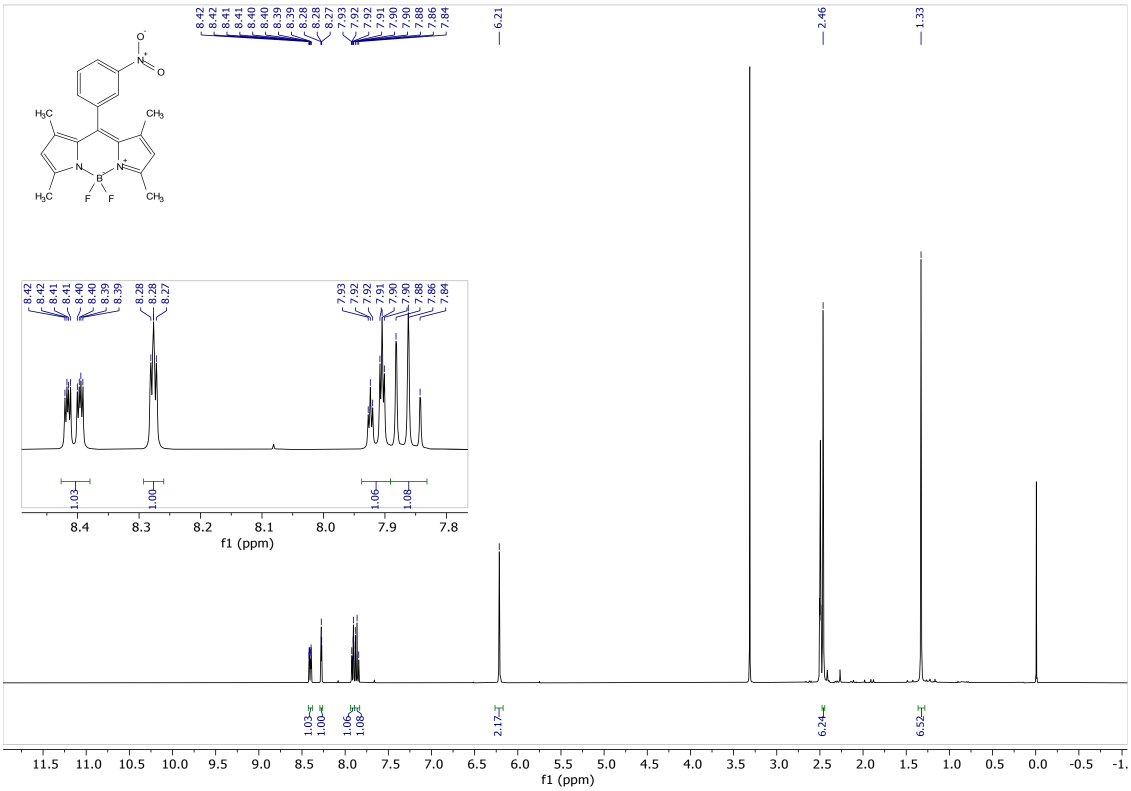

Compound **2** (DMSO-d_6)

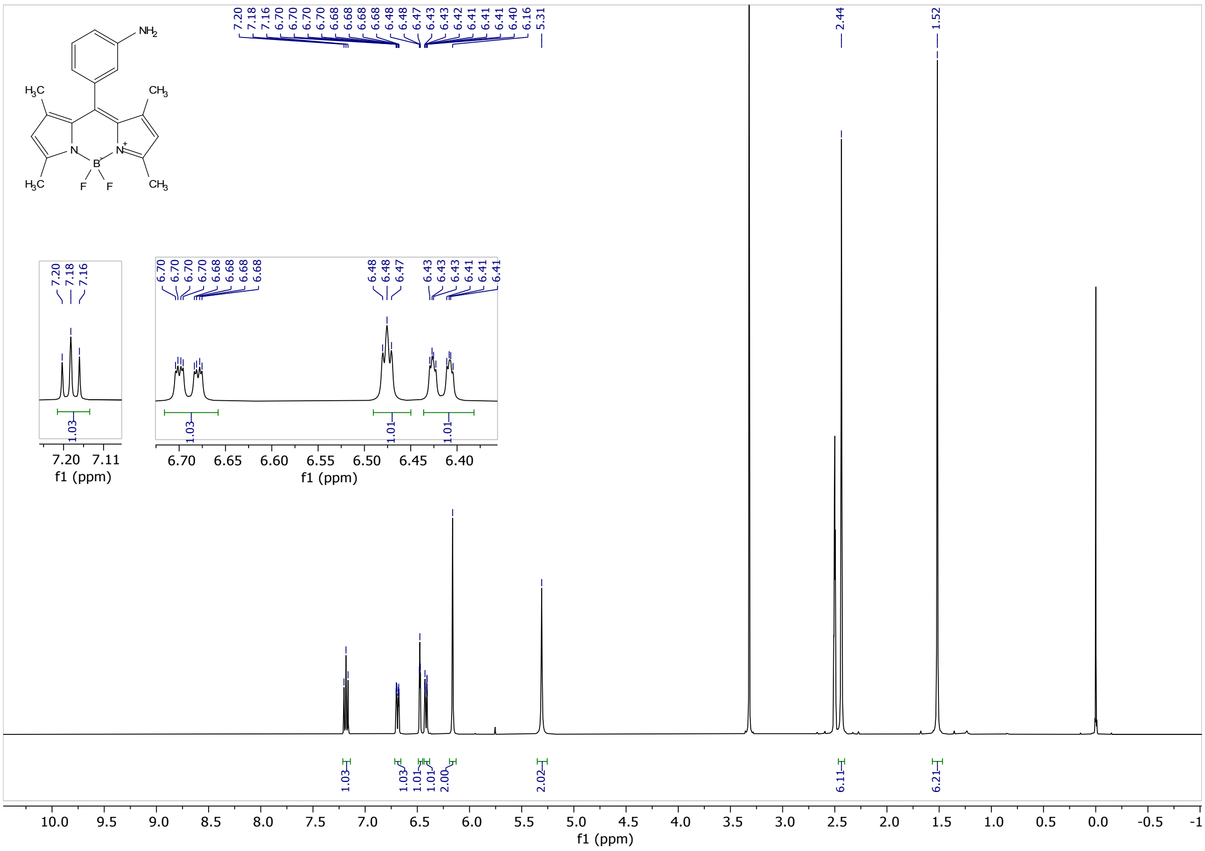

^{19}F-NMR for compound **2** (DMSO-d_6)

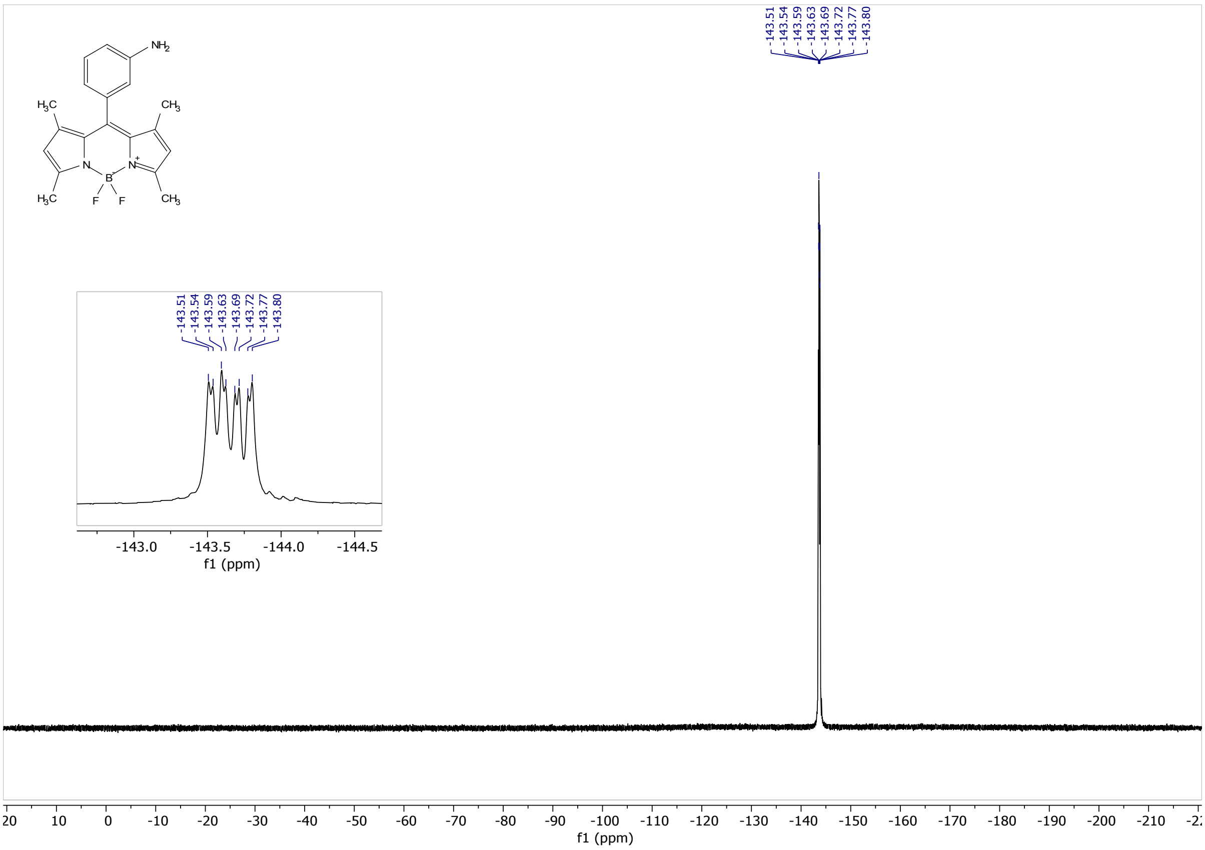

Compound **3** (CD_3CN)

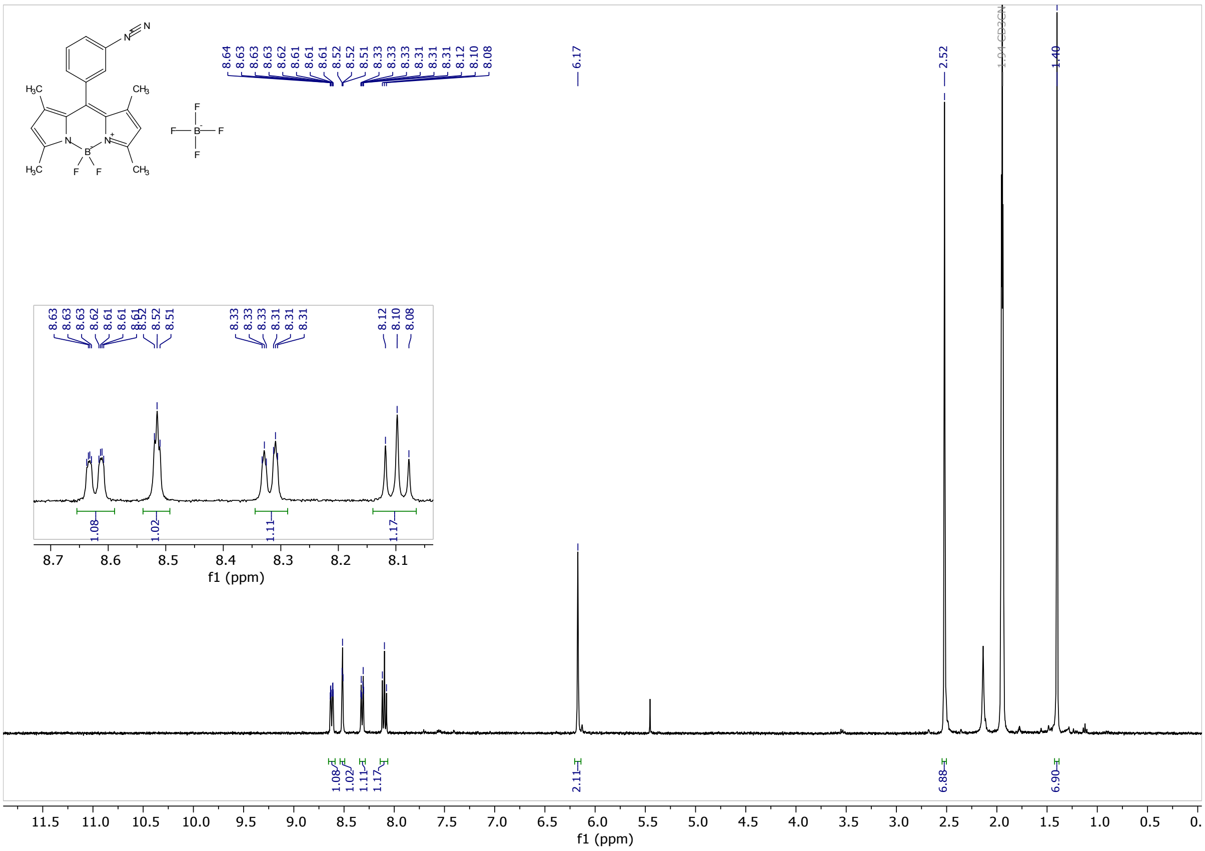

^{19}F-NMR for compound **3** (CD_3CN)

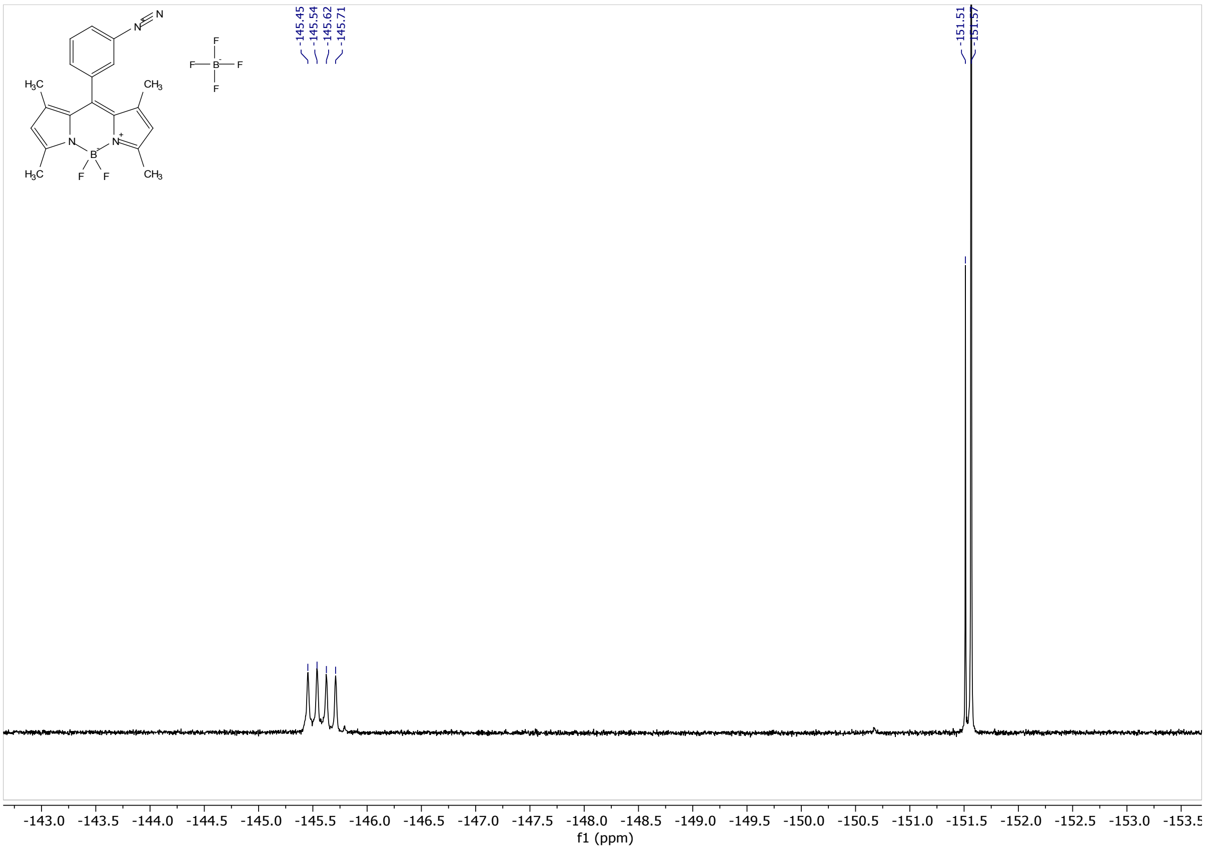

FT-IR for compound **3**

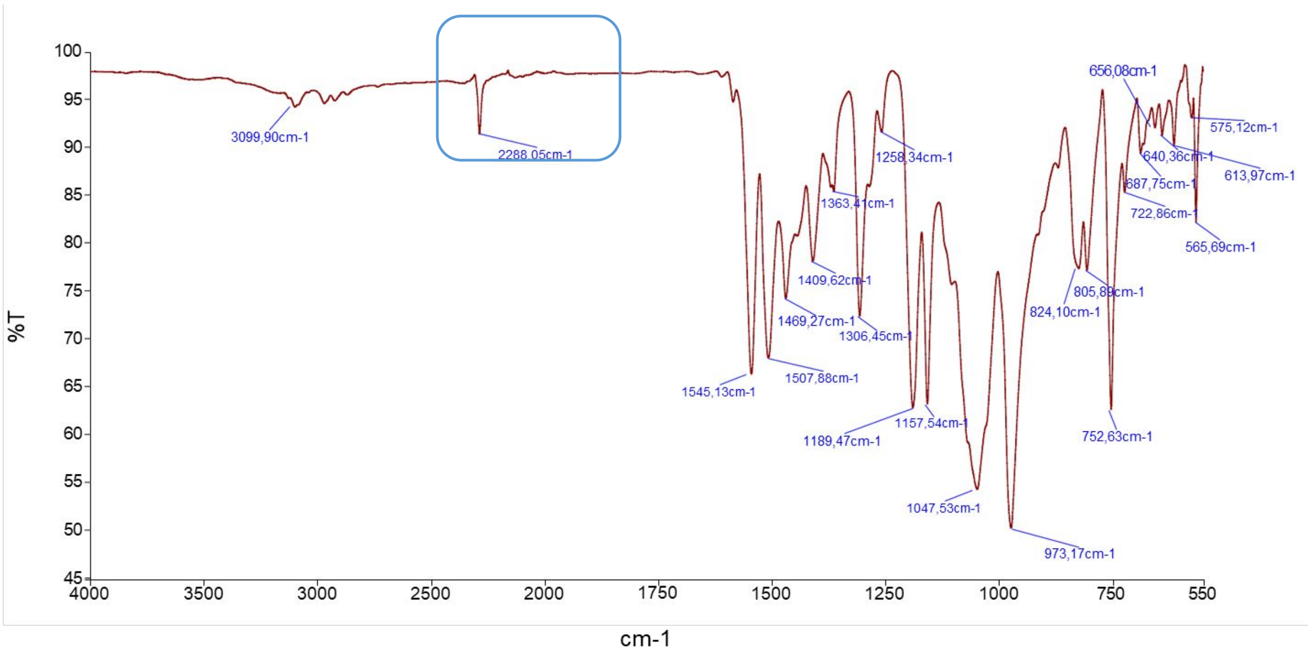

Compound **4** (DMSO-d_6)

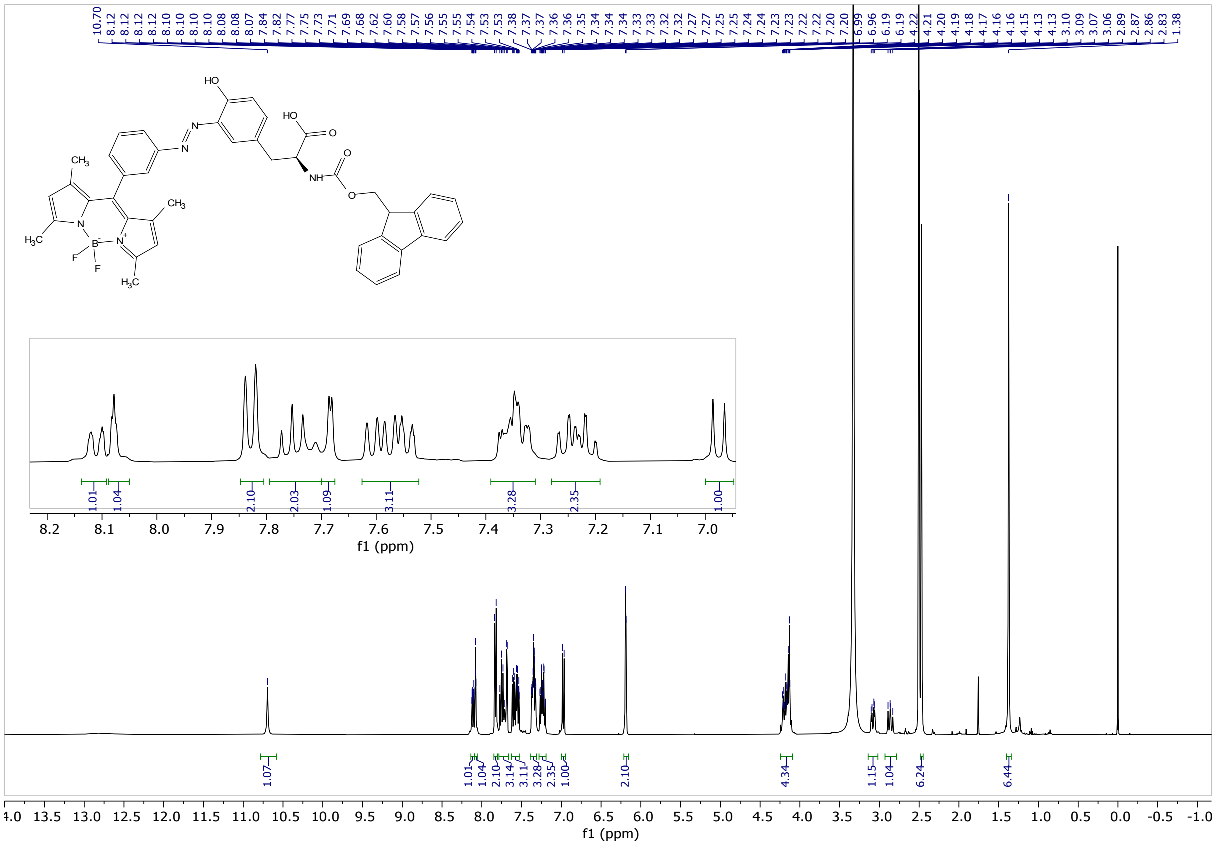

^{13}C-NMR for compound **4** (DMSO-d_6)

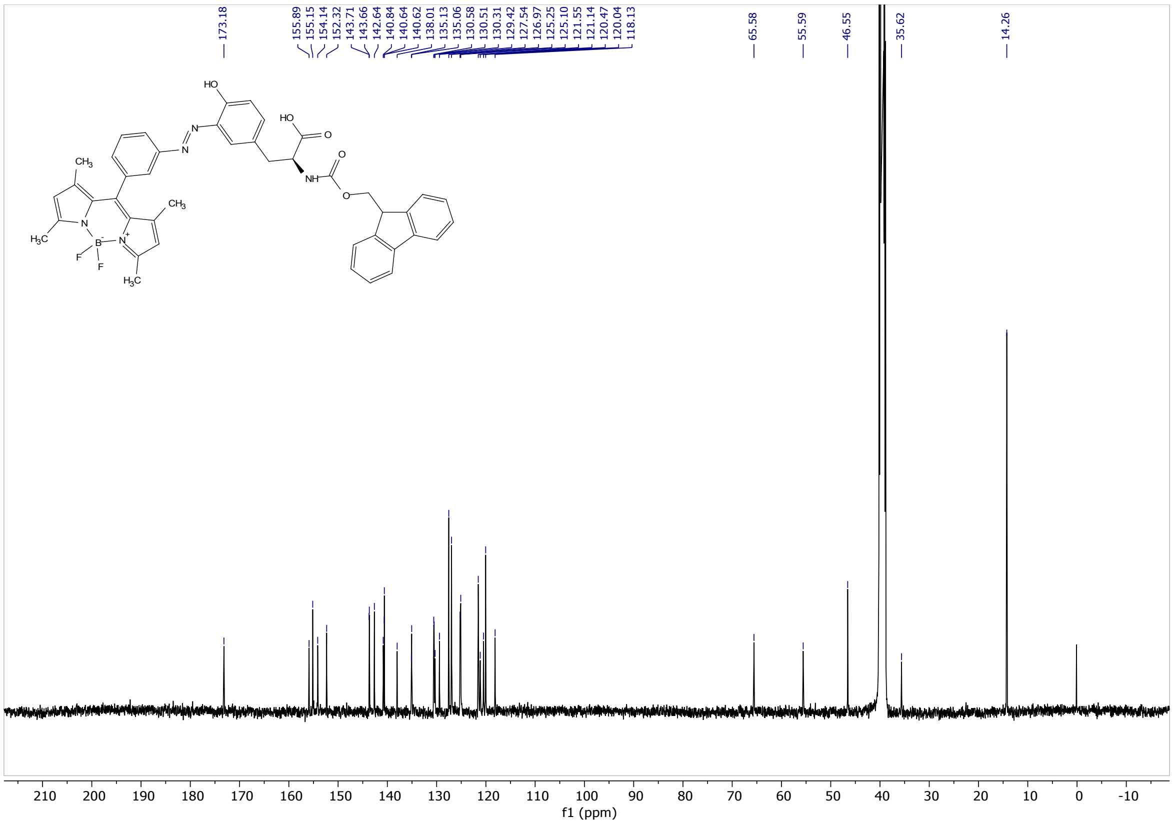

^{19}F-NMR for compound **4** (DMSO-d_6)

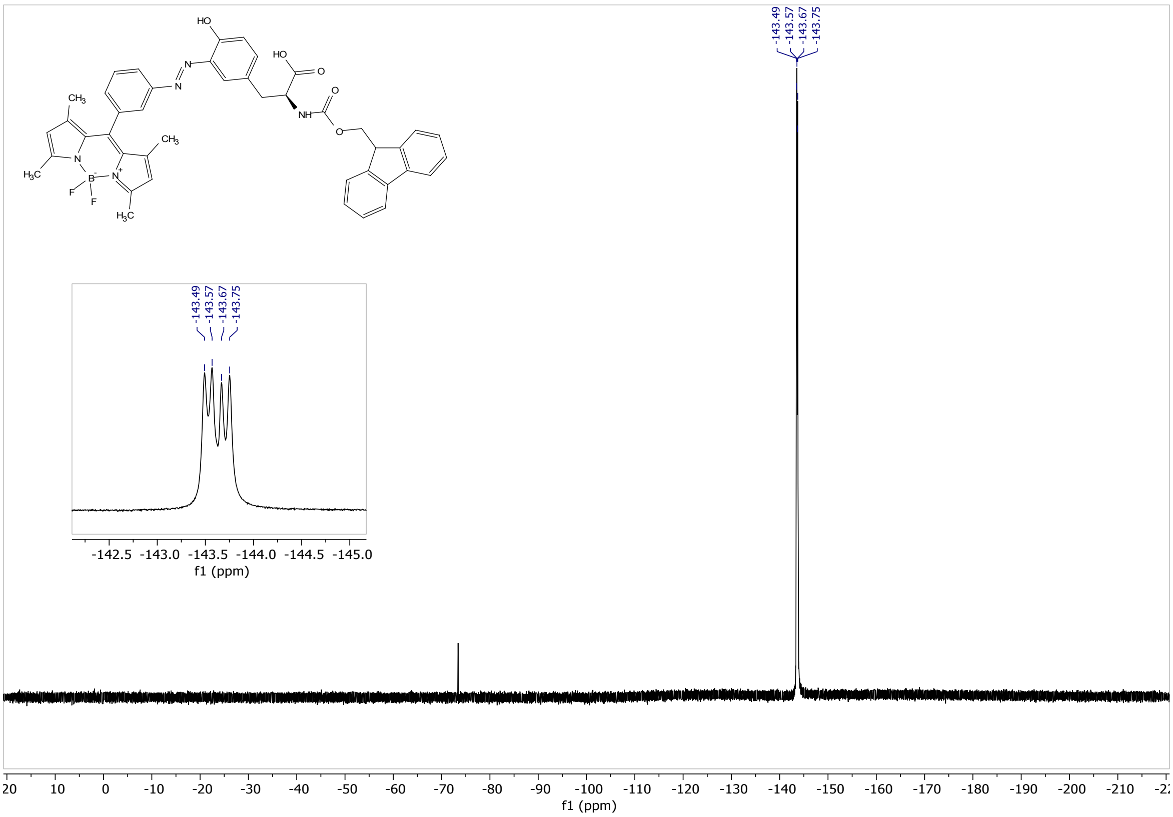

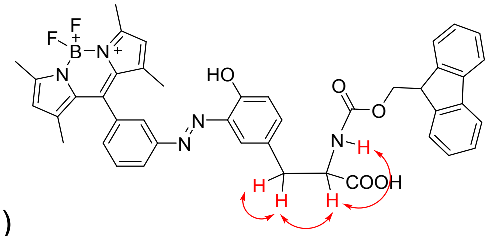

gCOSY for compound **4** (DMSO-d_6)

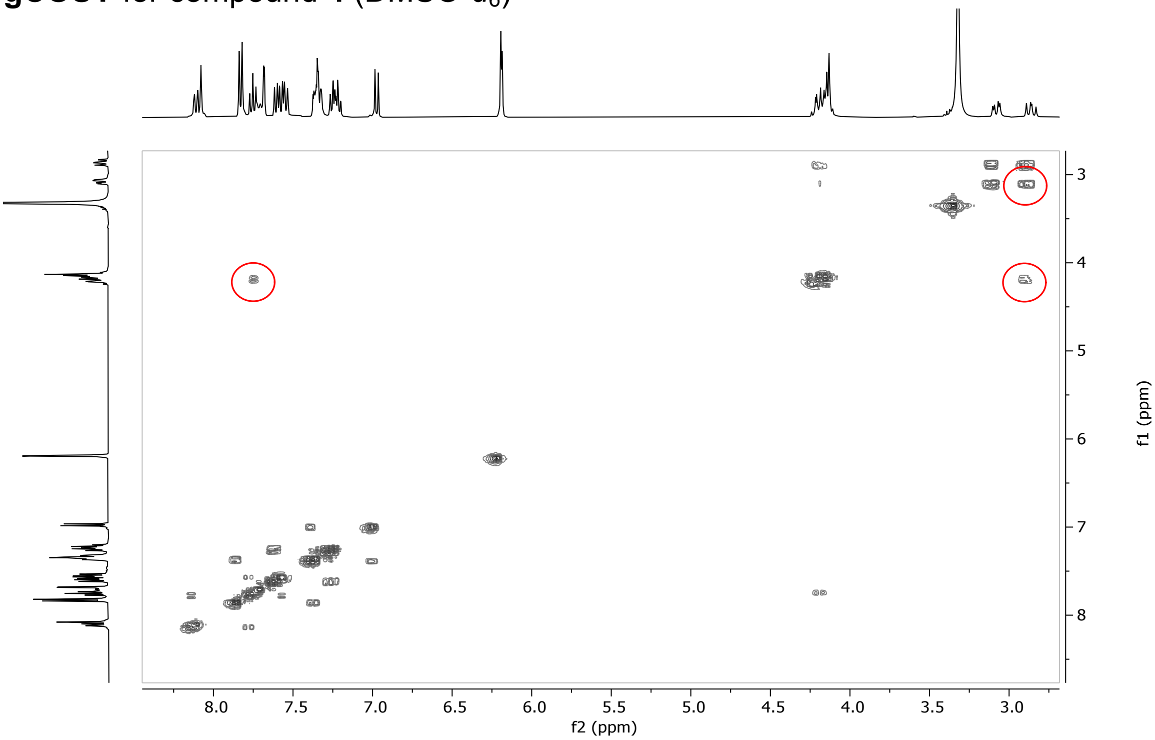

Compound **7** (DMSO-d_6)

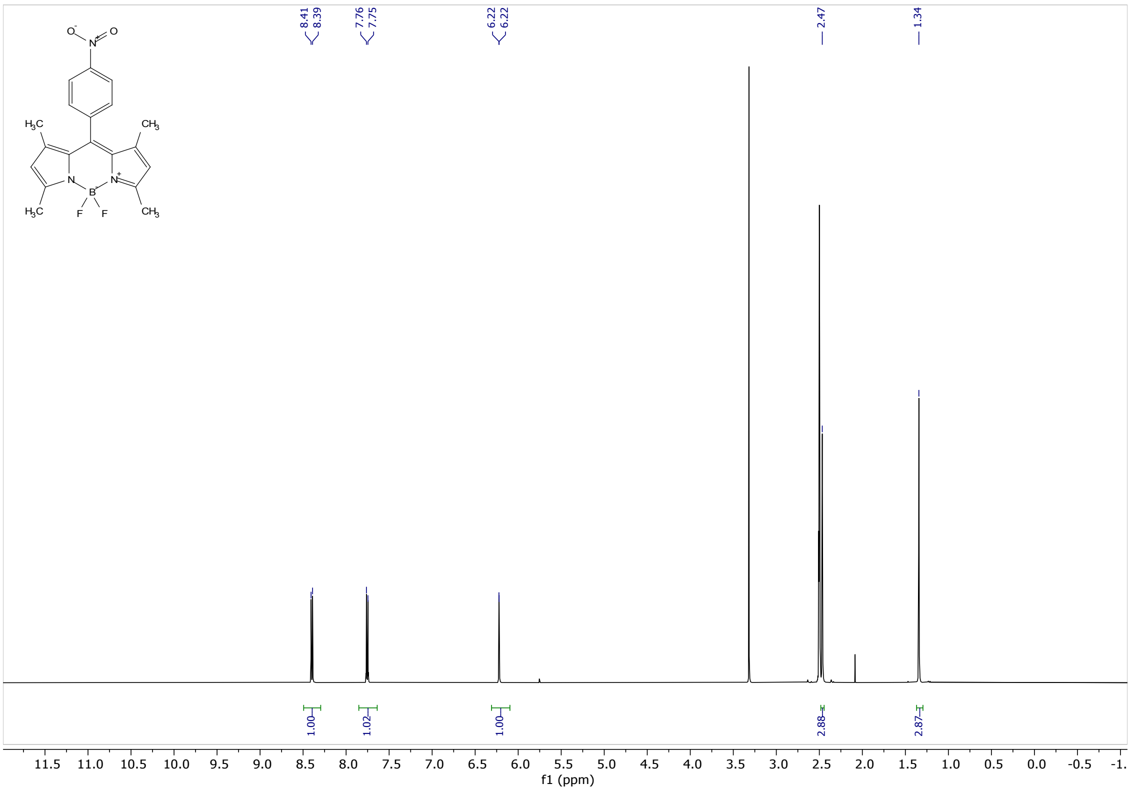

Compound **8** (DMSO-d_6)

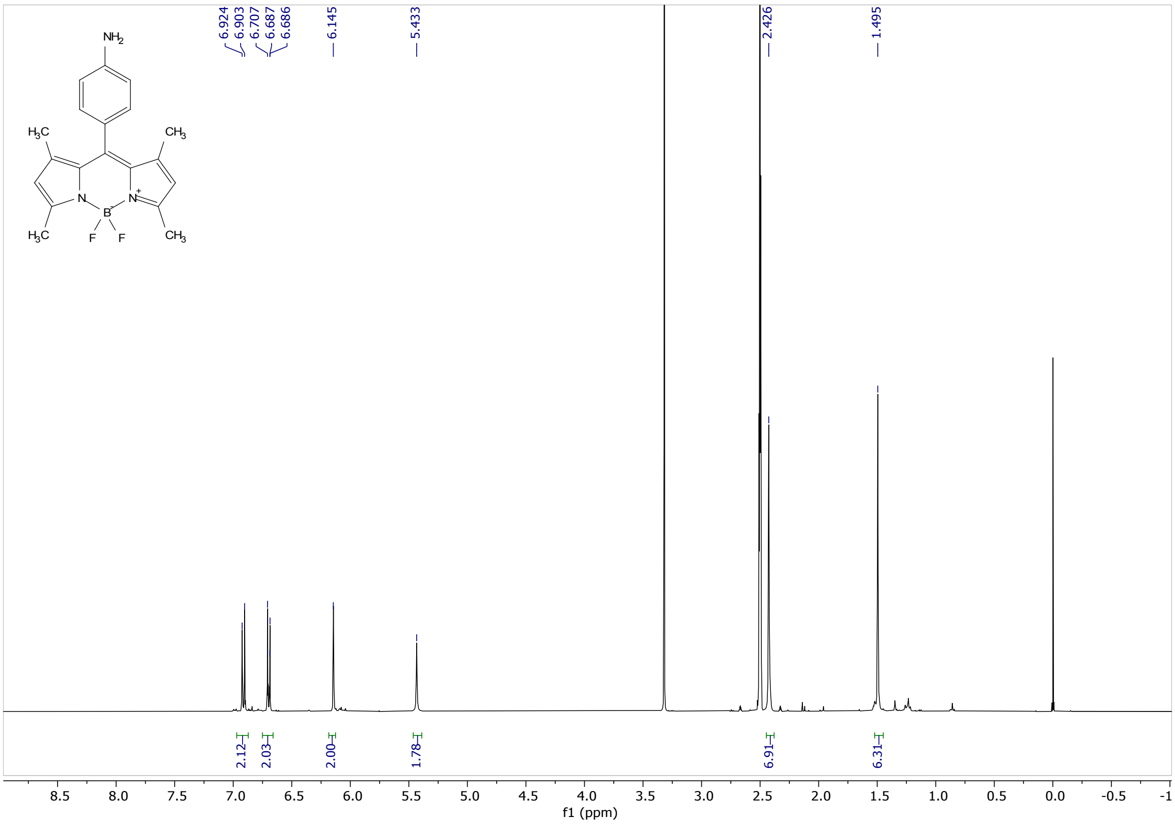

^{19}F-NMR for compound **8** (DMSO-d_6)

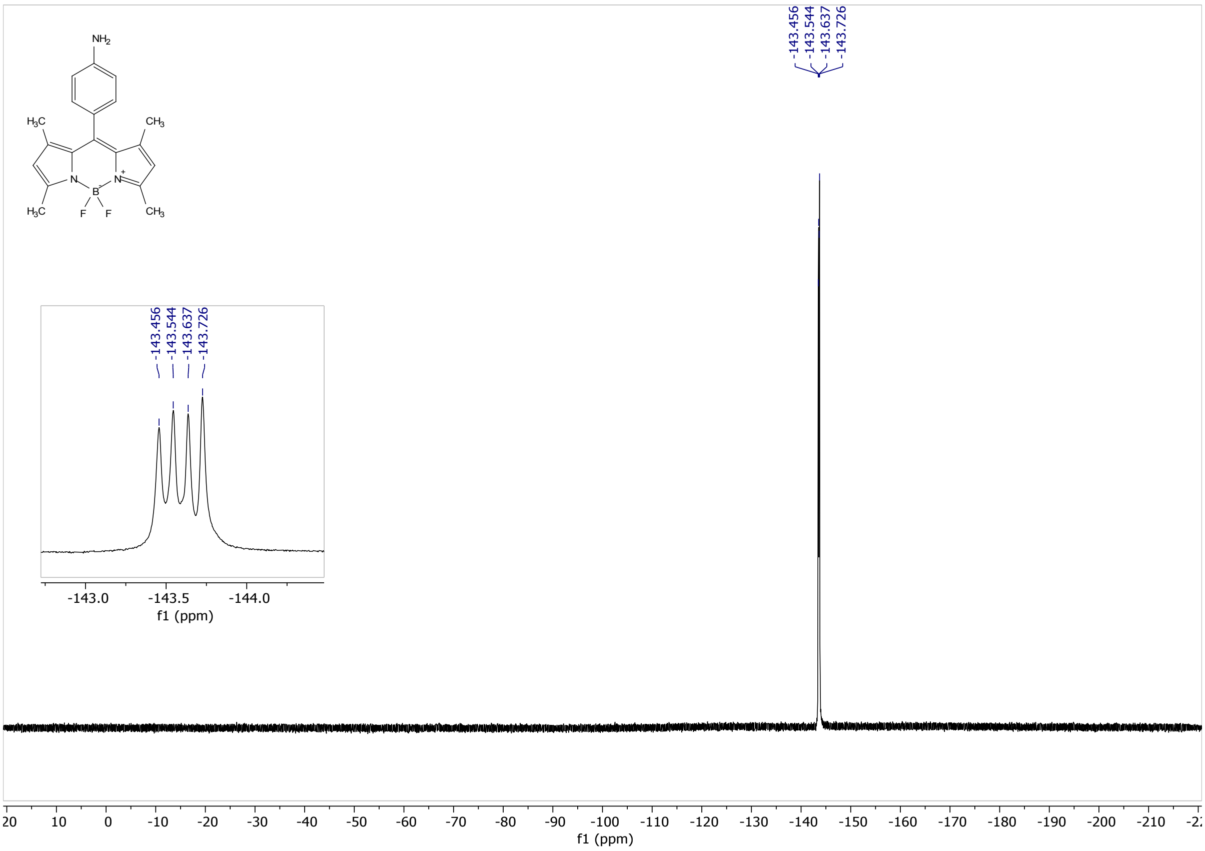

Compound **9** (DMSO-d_6)

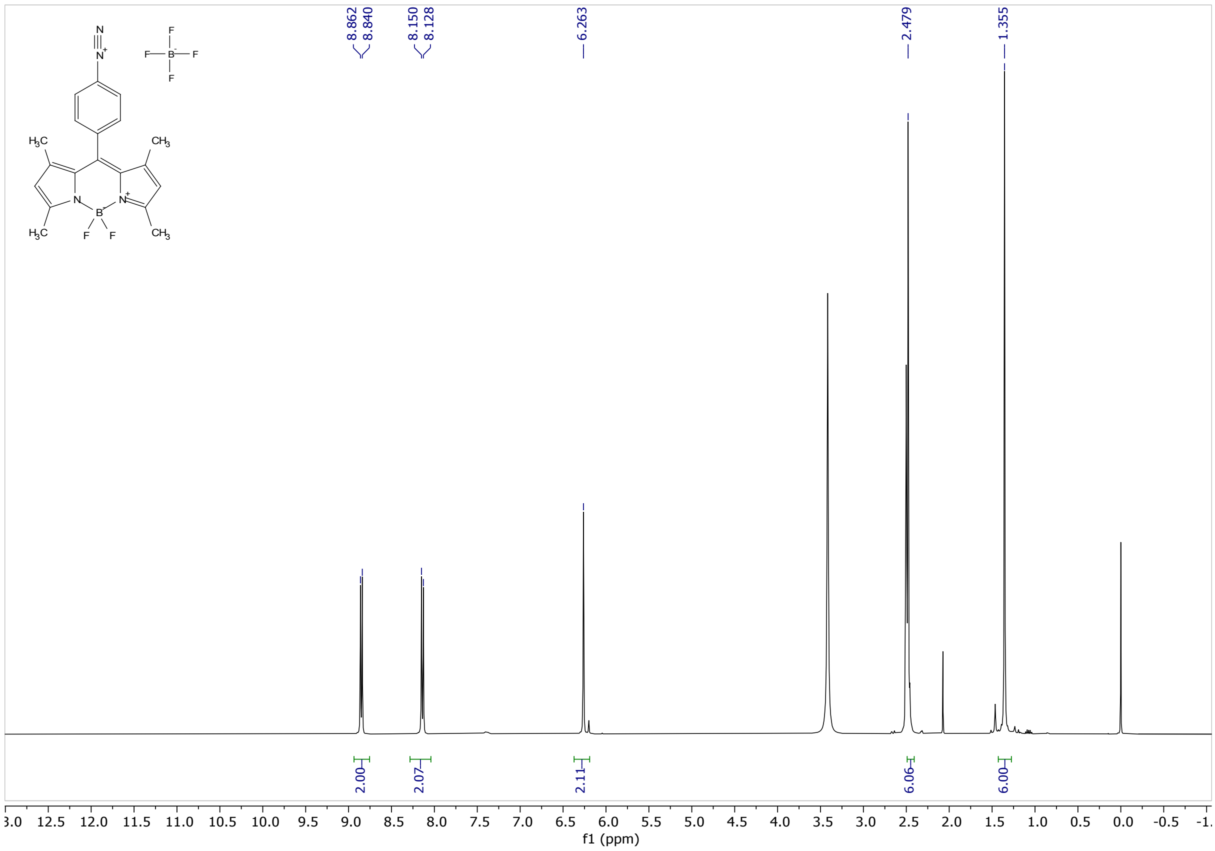

^{19}F-NMR for compound **9** (DMSO-d_6)

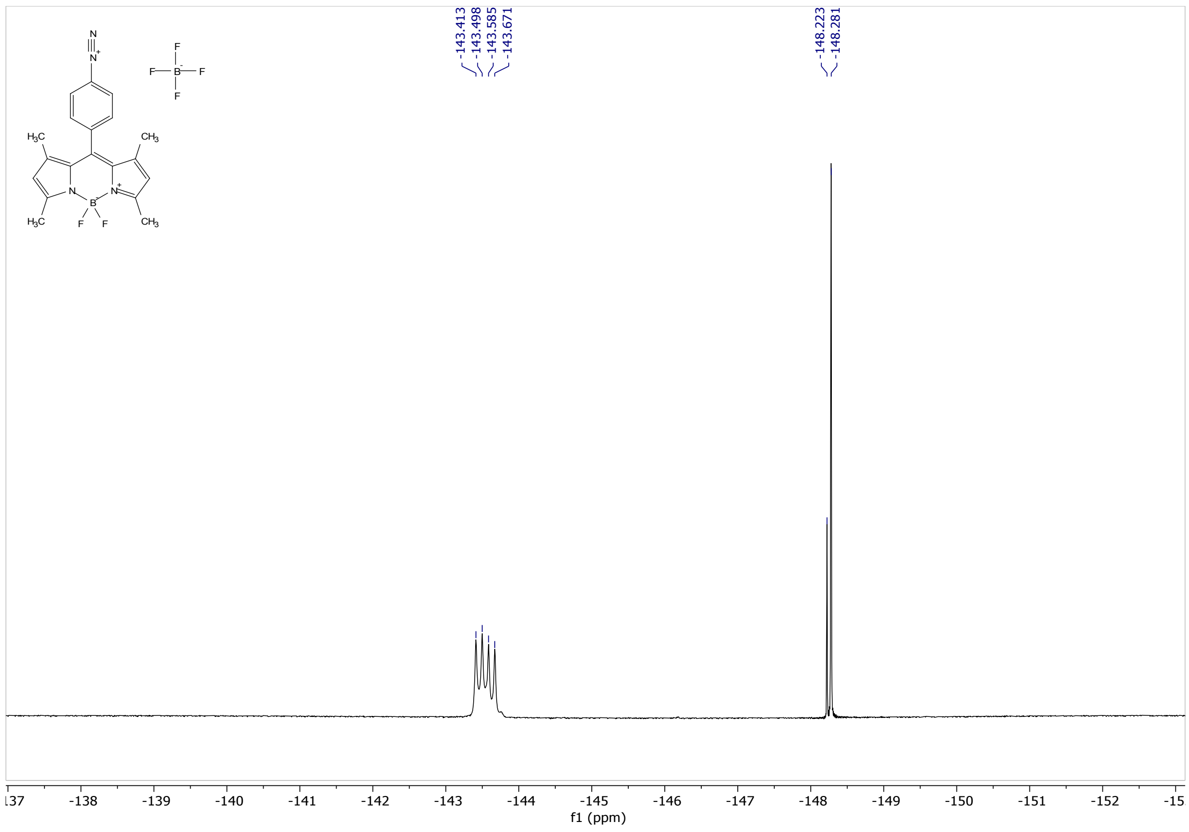

FT-IR for compound **9**

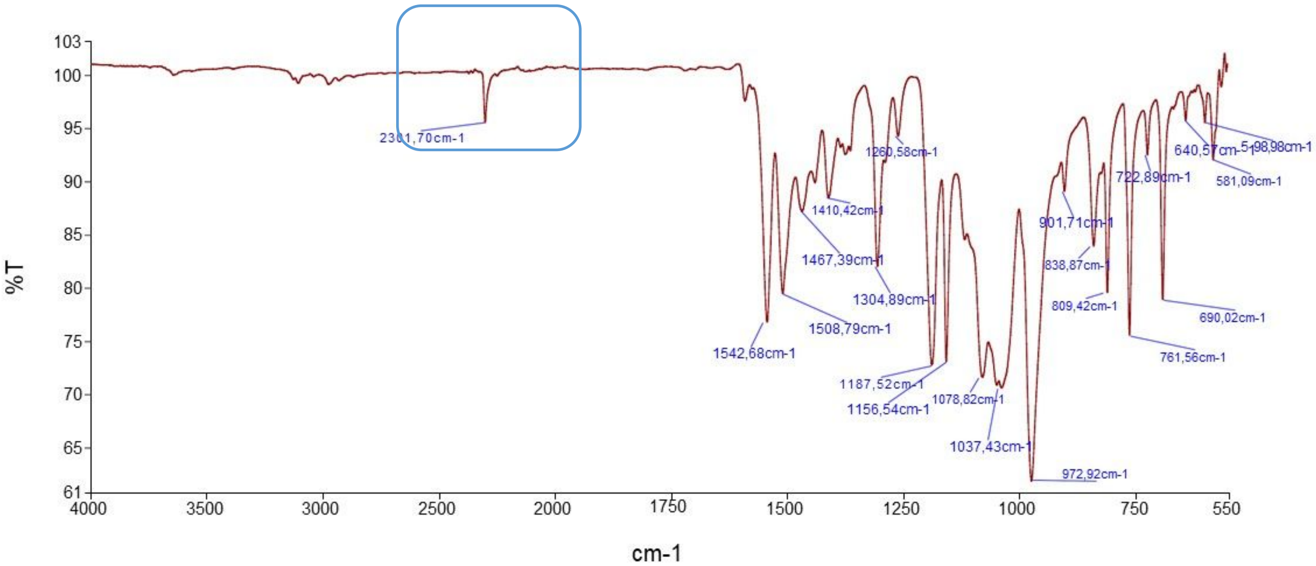

Compound **10** (DMSO-d_6)

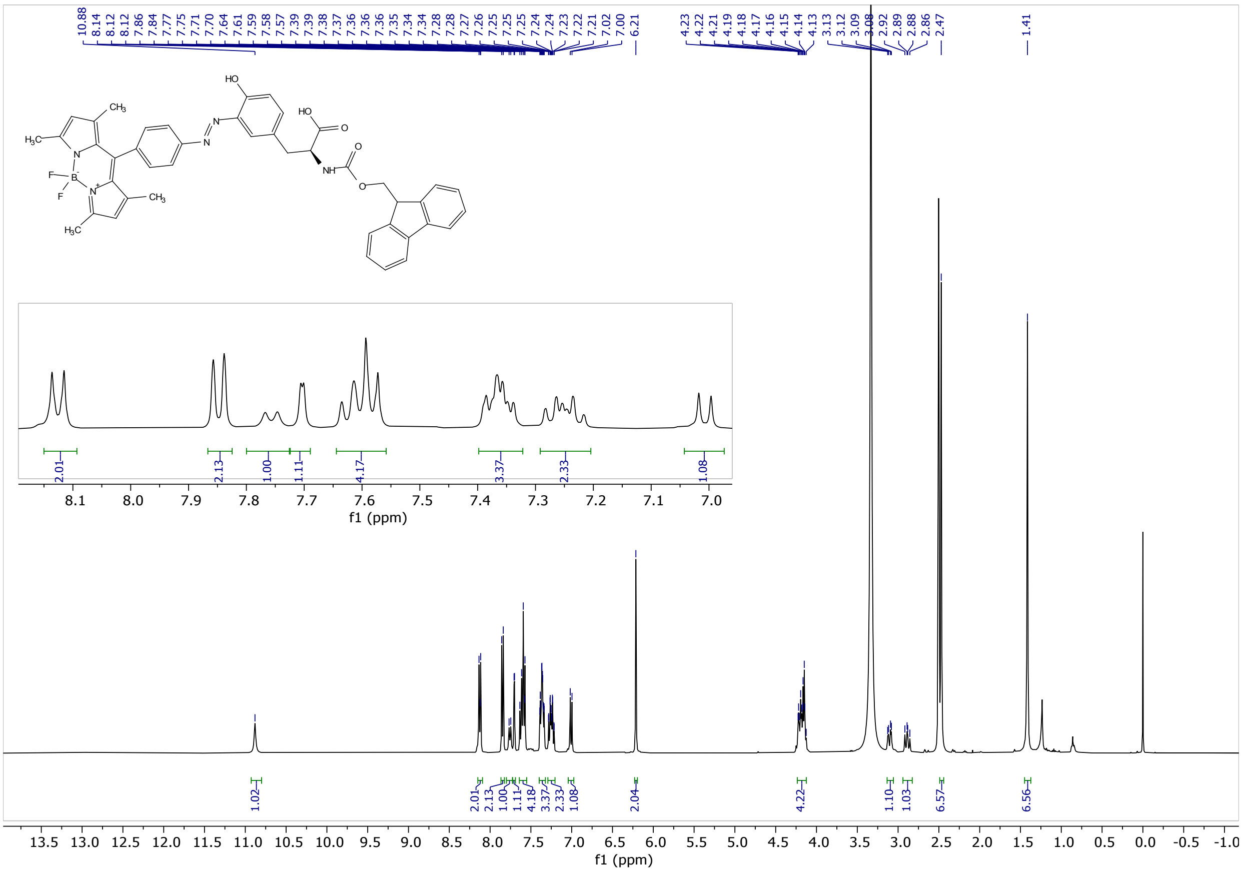

^{13}C-NMR for compound **10** (DMSO-d_6)

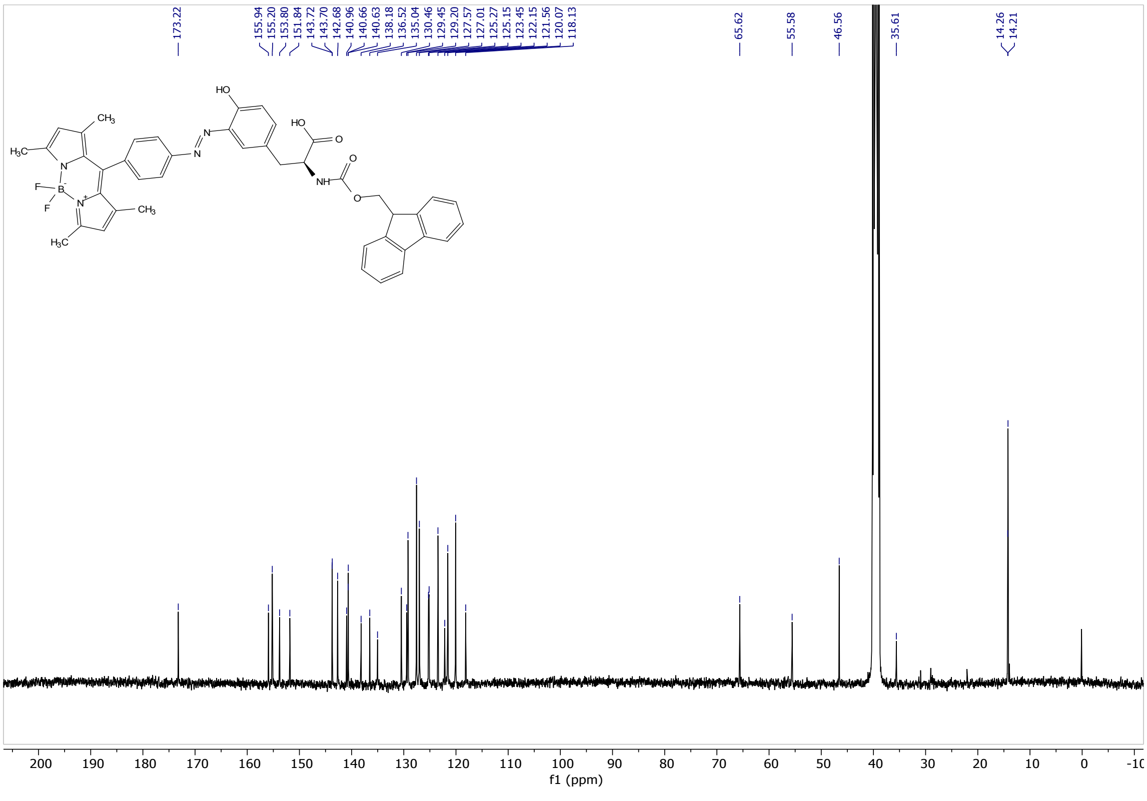

^{19}F-NMR for compound **10** (DMSO-d_6)

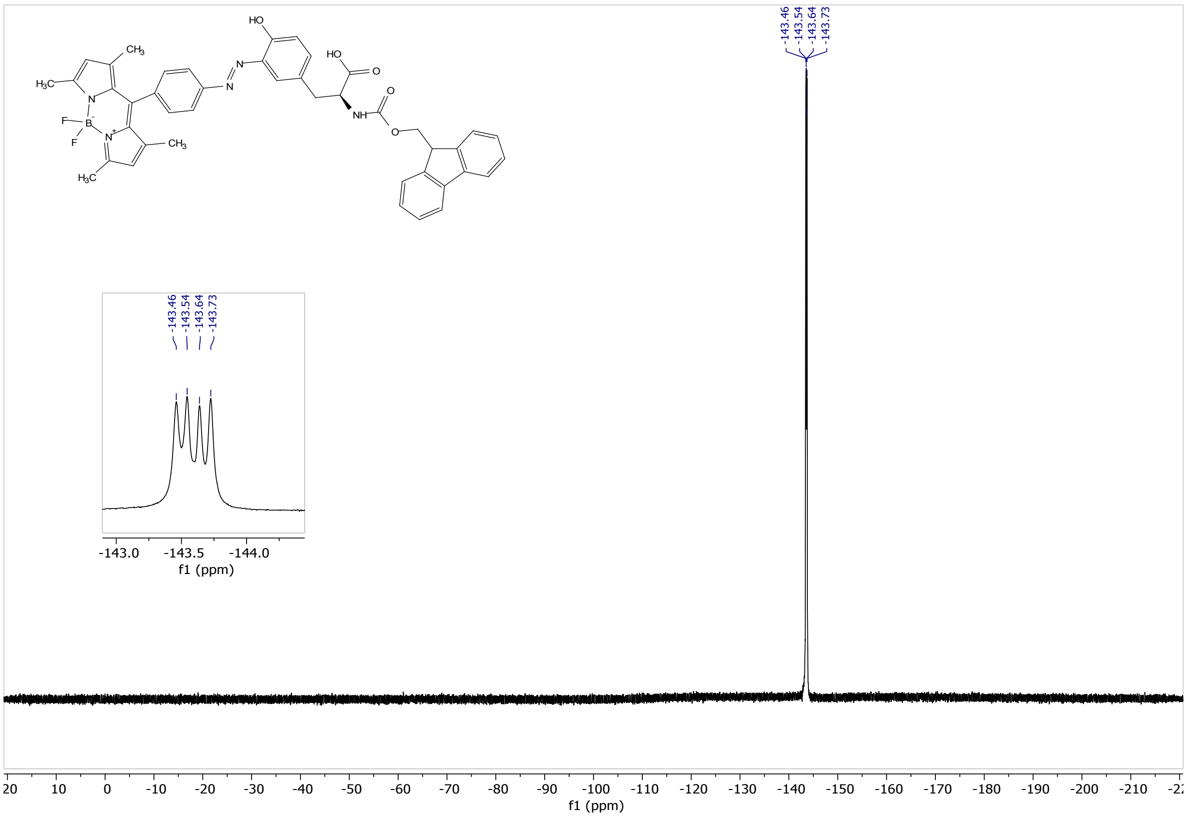

6. References

[1] Çeken, B.; Kízíl, M. Synthesis and DNA-cleaving activity of a series of substituted arenediazonium ions. *Russ. J. Bioorg. Chem.* **2008**, *34*, 488-498.

[2] Huo, Y.; Miao, J.; Li, Y.; Shi, Y.; Shi, H.; Guo, W. Aromatic primary monoamine-based fast-response and highly specific fluorescent probes for imaging the biological signaling molecule nitric oxide in living cells and organisms. *J. Mater. Chem. B* **2017**, *5*, 2483-2490.

[3] Tekdaş, D. A.; Viswanathan, G.; Topal, S. Z.; Looi, C. Y. W. F.; Tan, M. Y.; Zorlu, Y.; Gurek, A. G.; Lee, H. B.; Dumoulin, F. Antimicrobial activity of a quaternized BODIPY against Staphylococcus strains. *Org. Biomol. Chem.* **2016**, *14*, 2665-2670.

[4] Bridge, T.; Shaikh, S. A.; Thomas, P.; Botta, J.; McCormick, P. J.; Sachdeva, A. Site-specific encoding of photoactivity in antibodies enables light-mediated antibody–antigen binding on live cells. *Angew. Chem. Int. Ed.* **2019**, *58*, 17986-17993.
