# Supplementary material for: Nonperturbative Fluorogenic Labeling of Immunophilins Enables the Wash-free Detection of Immunosuppressants
Source: ACS Cent Sci. 2024 Mar 18;10(5):969–77. doi: 10.1021/acscentsci.3c01590 (PMC11117681; doi:10.1021/acscentsci.3c01590)
Supplement: Supplementary file 3 — oc3c01590_si_003.pdf [file oc3c01590_si_003.pdf]

oc-2023-01590k.R1

Name: Peer Review Information for "Non-perturbative fluorogenic labeling of immunophilins enables wash-free detection of immunosuppressants"

First Round of Reviewer Comments

Reviewer: 1

Comments to the Author

In the submitted manuscript, the authors described a tyrosine-specific labeling method to afford fluorogenic proteins for the detection of immunosuppressants. The authors first synthesized a diazonium BODIPY compound to attach to the phenol residue of tyrosine. The labeling specificity was optimized and confirmed by reacting with amino acids, short peptides and immunophilin proteins. Finally, the authors showed that engineered immunophilin with BODIPY labeled at the binding site is fluorogenic and can be used for wash-free detection of an immunosuppressive drug ligand. Overall, the experiments were well-performed, and the manuscript is well-written. I would recommend publication after addressing the following minor questions:

1. Since the spectra of azobenzene isomers overlap with BODIPY, could the authors comment on the cis/trans state of the Tyr-BODIPY adduct in the presence and absence of light and/or ligand?

2. The authors may consider citing other papers on optical imaging of immune systems such as Journal of the American Chemical Society, 2020, 142, 7075.

3. In the tacrolimus binding study, the authors conclude that the fluorogenicity is caused by enhanced hydrophobicity upon binding. Could it also be increased steric hindrance? In Figure 5d, the authors give the kinetic parameters for the binding. I wonder if the authors could measure the thermodynamic parameters using isothermal titration calorimetry, where hydrophobic interaction corresponds to enthalpic change, and restriction of fluorophore rotation corresponds to entropic change. This could help the reader understand the fluorogenic mechanism. If doing such experiments are not possible in the lab or for the current settings, the authors may consider citing references for discussion.

Reviewer: 2

Comments to the Author

The manuscript "Non-perturbative fluorogenic labeling of immunophilins enables wash-free detection of immunosuppressants" by Lavilla, Vendrella and co-workers disclose new diazonium BODIPY compounds for site-specific labeling of tyrosine residues in peptides via solid-phase synthesis as well as for late-stage functionalization of whole recombinant proteins. They demonstrate the application of these new fluorogenic compounds for immunophilin labelling. This manuscript will not only be of interest to the peptide synthesis and bioconjugation community, but also for the fields of imaging and immunobiology. i therefore strongly recommend publishing this work after addressing the following comments:

- to show the use in SPPS, the authors demonstrate the synthesis of a small fragment of amyloid beta, however, this peptide does not contain many amino acids that are often a concern (Cys, His, Met, Trp, Agr,..). Please demonstrate the labeling method on additional peptides and comment on the compatibility with the before-mentioned amino acids.

- along the same lines: are there any incompatibilities with native amino acids during the bioconjugation protocol that is described in this manuscript?

- the BODIPY connected via an azobenzene, which can likely be isomerized to its Z-confirmation by light irradiation. Does this switching change the binding behavior of tacrolimus to FKPB12 protein? And how does it affect the fluoroscence of the compound?

Author's Response to Peer Review Comments:

Formatting Needs

AU EMAIL: Please include the email address of the corresponding author on the first page of the manuscript, and the Supporting Information if submitted, with an asterisk next to their name in the author list. Please be sure to label “email.”

Answer: We have included the email addresses on the first pages of the manuscript and the Supporting Information.

SI PARAGRAPH: If the manuscript is accompanied by any supporting information for publication, a brief description of the supplementary material is required in the manuscript. The appropriate format is: Supporting Information. Brief statement in non-sentence format listing the contents of the material supplied as Supporting Information.

Answer: We have added a brief description of the supplementary material in the manuscript.

GENERAL REF FORMATTING: Periodical references should contain authors' surnames followed by initials, article title, journal abbreviation, year, volume number, and page range. Refs with more than 10 authors should list the first 10 and then be followed by "et al."

Answer: We have amended the references as required.

SI HEADER: The supporting information should be formatted with a cover sheet listing authors, author affiliations, corresponding author email, manuscript title, and the number of pages, figures, and tables. The Author affiliations must match the MS.

Answer: We have included the cover sheet in the revised Supporting Information.

SI PG#S: The supporting information pages must be numbered consecutively, starting with page S1.

Answer: We have amended this point in the revised Supporting Information.

Response to Reviewers

Reviewer 1

1. Since the spectra of azobenzene isomers overlap with BODIPY, could the authors comment on the cis/trans state of the Tyr-BODIPY adduct in the presence and absence of light and/or ligand?

Answer: We thank the reviewer for the comment. The cis/trans photoisomerization of azobenzenes structures has been extensively reported in the literature.[1-3] Therefore, it is likely that azobenzene-containing BODIPY adducts will exhibit some degree of cis/trans isomerization upon irradiation. We have clarified this point and added new references to the revised manuscript (new references 54-56).

2. The authors may consider citing other papers on optical imaging of immune systems such as Journal of the American Chemical Society, 2020, 142, 7075.

Answer: We thank the reviewer for the suggestion and included this reference in the revised manuscript (new reference 17).

3. In the tacrolimus binding study, the authors conclude that the fluorogenicity is caused by enhanced hydrophobicity upon binding. Could it also be increased steric hindrance? In Figure 5d, the authors give the kinetic parameters for the binding. I wonder if the authors could measure the thermodynamic parameters using isothermal titration calorimetry, where hydrophobic interaction corresponds to enthalpic change, and restriction of fluorophore rotation corresponds to entropic change. This could help the reader understand the fluorogenic mechanism. If doing such experiments are not possible in the lab or for the current settings, the authors may consider citing references for discussion.

Answer: We thank the reviewer for the insight. Indeed, it is well known that the fluorogenic behavior of BODIPY systems can be attributed to entropic factors, in which an increased steric hindrance upon binding promotes the restriction of the BODIPY rotation along the phenyl coordinate leading to decrease of nonradiative decay.[4] Therefore, it is plausible that entropic changes may contribute to the fluorogenicity mechanism. BODIPY fluorophores have been also reported as turn-on polarity sensors, where hydrophobic interactions with target proteins can lead to fluorescence enhancements.[5] We have clarified this point about the possible steric hindrance contribution to the fluorogenicity mechanism including references in the revised manuscript (new references 51 and 52).

References

[1] Morstein, J.; Romano, G.; Hetzler, B. E.; Plante, A.; Haake, C.; Levitz, J.; Trauner, D. *Angew. Chem. Int. Ed.* **2022**, *61*, e202117094.

[2] Albert, L.; Nagpal, J.; Steinchen, W.; Zhang, L.; Werel, L.; Djokovic, N.; Ruzic, D.; Hoffarth, M.; Xu, J.; Kaspareit, J.; et.al. *ACS Cent. Sci.* **2022**, *8*, 57-66.

[3] Crespi, S.; Simeth, N. A.; B. König, N. A. *Nat. Rev. Chem.* **2019**, *3*, 133-146.

[4] Lee, S.; Heo, J.; Woo, H. C.; Lee, J.-A.; Seo, Y. H.; Lee, C.-L.; Kim, S.; Kwon, O.-P. *Chem. Eur. J.* **2018**, *24*, 13706-13718.

[5] Dorh, N.; Zhu, S.; Dhungana, K. B.; Pati, R.; Luo, F.-T.; Liu, H.; Tiwari, A. *Sci. Rep.* **2015**, *5*, 18337.

Reviewer 2

1. To show the use in SPPS, the authors demonstrate the synthesis of a small fragment of amyloid beta, however, this peptide does not contain many amino acids that are often a concern (Cys, His, Met, Trp, Agr,..). Please demonstrate the labeling method on additional peptides and comment on the compatibility with the before-mentioned amino acids.

Answer: We appreciate the reviewer's comment. The potential for other amino acids, such as His or Trp, to interfere in azo couplings is well documented and important consideration.[6] However, in the context of protein labeling (e.g., in this case, immunophilins PPIA and FKBP12), these reactions preferentially occur at Tyr residues due to their exposure on the surface of most proteins. This results in preferential labeling of exposed Tyr over hindered Trp and His.[7] Our results in Figure 4 demonstrate such specificity, supported by mass spectrometry analysis. We have included this consideration in the revised manuscript (references 35 and 44 in the manuscript).

2. Along the same lines: are there any incompatibilities with native amino acids during the bioconjugation protocol that is described in this manuscript?

Answer: The mass spectrometry analysis in Figures 4, S9 and S10 demonstrate that BODIPY labels Tyr residues and that no conjugation to other hydrophobic amino acids was detected. We believe that these experiments provide robust evidence of the preferential reactivity of the diazonium BODIPY with tyrosine residues during bioconjugation.

3. The BODIPY connected via an azobenzene, which can likely be isomerized to its Zconfirmation by light irradiation. Does this switching change the binding behavior of tacrolimus to FKPB12 protein? And how does it affect the fluorescence of the compound?

Answer: We thank the reviewer for the comments. We performed SPR experiments to compare the binding affinity of BODIPY-labeled FKBP12 Y26F Y80F its non-labeled counterpart. As shown in Figure 5, we obtained very similar K_D values for both, in the 30-40 nM range. These results indicate that the presence of an azobenzene-BODIPY does not significantly affect the binding to tacrolimus. We agree with the reviewer that, based on the extensive reported literature on other azobenzenes, it is possible that non-radiative cis/trans photoisomerizations may occur. Previous reports have indicated that suppressing photoisomerization or reducing the efficiency of other non-radiative transitions (e.g., by sterically restricting the molecular movement required for isomerization) could enhance the fluorescence of these compounds.[8] We have included this consideration in the revised manuscript and added new references (refs. 53-56 in the manuscript).

References

[6] Gavrilyuk, J.; Ban, H.; Nagano, M.; Hakamata, W.; Barbas III, C. F. *Bioconjug. Chem.* **2012**, *23*, 2321-2328.

[7] Sengupta, S.; Chandrasekaran, S. *Org. Biomol. Chem.* **2019**, *17*, 8308-8329.

[8] Xue, L.; Pan, Y.; Zhang, S.; Chen, Y.; Yu, H.; Yang, Y.; Mo, L.; Sun, Z.; Li, L.; Yang, H. *Crystals* **2021**, *11*, 840.

oc-2023-01590k.R2

Name: Peer Review Information for "Non-perturbative fluorogenic labeling of immunophilins enables wash-free detection of immunosuppressants"

Second Round of Reviewer Comments

Reviewer: 1

Comments to the Author

The authors have properly addressed the questions and it is ready to be published.

Reviewer: 2

Comments to the Author

The manuscript "Non-perturbative fluorogenic labeling of immunophilins enables wash-free detection of immunosuppressants" by Lavilla, Vendrella and co-workers disclose new diazonium BODIPY compounds for site-specific labeling of tyrosine residues in peptides via solid-phase synthesis as well as for late-stage functionalization of whole recombinant proteins. They demonstrate the application of these new fluorogenic compounds for immunophilin labelling. This manuscript will not only be of interest to the peptide synthesis and bioconjugation community, but also for the fields of imaging and immunobiology.

The authors of this manuscript thoroughly addressed all comments raised by this reviewer, and added new sections and references to their manuscript. I therefore strongly support accepting this

manuscript for publication in ACS Central Science and congratulate the authors on their excellent results.

Author's Response to Peer Review Comments:

Formatting needs (ie individual references) addressed in the revised manuscript.
